# Increasing prevalence of hot drought across western North America since the 16th century

**DOI:** 10.1126/sciadv.adj4289

**Published:** 2024-01-24

**Authors:** Karen E. King, Edward R. Cook, Kevin J. Anchukaitis, Benjamin I. Cook, Jason E. Smerdon, Richard Seager, Grant L. Harley, Benjamin Spei

**Affiliations:** ^1^ Department of Geography and Sustainability, University of Tennessee, Knoxville, 1000 Phillip Fulmer Way, Knoxville, TN 37996, USA.; ^2^ Tree Ring Laboratory, Lamont-Doherty Earth Observatory of Columbia University, 61 Route 9W, Palisades, NY 10964, USA.; ^3^ School of Geography, Development, and Environment, University of Arizona, 1064 Lowell Street, Tucson, AZ 85721, USA.; ^4^ Laboratory of Tree-Ring Research, University of Arizona, 1215 E Lowell Street, Tucson, AZ 85721, USA.; ^5^ NASA Goddard Institute for Space Studies, 2880 Broadway, New York, NY 10025, USA.; ^6^ Ocean and Climate Physics Division, Lamont-Doherty Earth Observatory of Columbia University, 61 Route 9W, Palisades, NY 10964, USA.; ^7^ Columbia Climate School, Columbia University, New York, NY 10027, USA.; ^8^ Department of Earth and Spatial Sciences, University of Idaho, 875 Perimeter Drive MS3021, Moscow, ID 83843, USA.; ^9^ Department of Forest, Rangeland, and Fire Sciences, University of Idaho, 975 West 6th Street, Moscow, ID 83843, USA.

## Abstract

Across western North America (WNA), 20th-21st century anthropogenic warming has increased the prevalence and severity of concurrent drought and heat events, also termed hot droughts. However, the lack of independent spatial reconstructions of both soil moisture and temperature limits the potential to identify these events in the past and to place them in a long-term context. We develop the Western North American Temperature Atlas (WNATA), a data-independent 0.5° gridded reconstruction of summer maximum temperatures back to the 16th century. Our evaluation of the WNATA with existing hydroclimate reconstructions reveals an increasing association between maximum temperature and drought severity in recent decades, relative to the past five centuries. The synthesis of these paleo-reconstructions indicates that the amplification of the modern WNA megadrought by increased temperatures and the frequency and spatial extent of compound hot and dry conditions in the 21st century are likely unprecedented since at least the 16th century.

## Teaser

Trees document the strongest association between summer temperature and drought in recent decades compared to past five centuries.

## INTRODUCTION

Over the past century, anthropogenic climate change has increased the frequency and intensity of concurrent heat and drought events globally (*1**–**4*). In a warming climate, a more comprehensive understanding of compound climate hazards has immediate relevance for evaluating and planning for climate change impacts. Individual climatic events can have serious effects on agriculture, infrastructure, and ecosystems, but compounding hazards can result in cascading and intensified consequences for these systems (*5*, *6*). The combination of anomalous heat with rainfall deficits have already led to droughts that are substantially more intense because of high temperatures and elevated vapor pressure deficits and have thus been called hot droughts (*7*, *8*). While the past century documents an increase in the concurrence of heat and drought conditions around the world, the mechanism for this is still somewhat unclear. For example, one explanation for the increase in hot drought is that because droughts are now occurring in a warmer world, the probability of any given event occurring during a warm year is increasing (*9*). Another explanation suggests that temperature has an amplifying effect on drought through increased evaporative demand (*10*, *11*). In addition to uncertainties related to the mechanisms driving the 20th-21st century increases in hot drought occurrence, little is known about the pre-instrumental frequency and magnitude of compound hot and dry conditions. While the paleorecord allows for the longer-term evaluation and contextualization of modern climate extremes, the utility of paleorecords to document changes in shorter-term extremes and to assess compound extreme events (*12*, *13*) is still unexplored. However, the systematic collection and analysis of paleoclimate records can indeed provide evidence of these changes over longer timescales (*14*).

As in many other regions around the world, both instrumental records and model-based attribution establish unequivocally increasing temperature trends for western North America (WNA), as well as increasing coeval extreme heat and drought events during the 20th and 21st centuries (*15**–**19*). At the same time, there is no evidence of anthropogenic trends in precipitation over the past decades in WNA (*20*, *21*), although some have argued for limited evidence of anthropogenic spring season drying in the south of southwestern North America and summer drying in the Pacific Northwest (*22*). While the local- to regional-scale occurrence of hot drought in WNA has been documented since the turn of the 20th century [e.g., the Dust Bowl Drought of 1932 to 1938 (*23*)], beginning in the early 21st century, WNA experienced a particularly severe, protracted, and spatially extensive hot drought. Across the region, the first two decades of the 21st century were the driest 22-year period over the past 1200 years, with an average soil moisture anomaly only analogous to the 22 years of the late 16th-century megadrought (*10*, *21*, *24*). Because of both its duration and severity, the 21st-century WNA drought is also considered a megadrought (*21*, *24*). However, the occurrence of megadrought conditions is not a unique feature of the 21st century in WNA (*25*). While 21st-century precipitation deficits alone would have been sufficient to sustain drought conditions across WNA (*10*), the additional contributions of anthropogenic climate change through temperature-driven increases in evapotranspiration (*26*) and vapor pressure deficits (*27*) exacerbated soil moisture deficits and increased both the spatial extent and the duration to levels characteristic of megadrought conditions (*10*, *24*).

While all evidence therefore suggests that the modern megadrought across much of WNA is an anthropogenically influenced hot drought, (*10*, *15*, *16*, *24*, *28*, *29*), the relationship between temperature and the severity and persistence of past droughts in this region remains almost entirely unknown before the instrumental era. This stems from the comparative paucity of temperature-sensitive tree-ring chronologies in the coterminous United States (*30**–**33*) despite the region having comprehensive paleoclimate evidence of hydroclimate variability over the past millennium (*25*). Furthermore, existing temperature reconstructions (*34**–**36*) make use of some of the same drought-sensitive trees used for soil moisture reconstructions and, thus, do not allow for an independent separation of temperature and drought through time.

Providing paleoclimatic context for modern temperature extremes, trends, and their role in drought severity is critical for better understanding the range of possible hot drought intensities, the drivers of these compound events, and for anticipating the potential consequences of hot drought under a range of future emission scenarios. Here, we synthesize a network of tree-ring density and blue intensity (BI) data (see the Supplementary Materials) from across WNA to develop a 0.5° spatial field reconstruction of summer maximum temperatures (June to August; JJA *T*_max_) that is independent of available drought reconstructions and herein referred to as the Western North American Temperature Atlas (WNATA). Over the period 1553 to 2020 CE, we use the WNATA in combination with a preexisting gridded drought reconstruction (*37*) for an examination of how modern temperature-drought relationships compare to those documented over the past ~480 years, particularly during past megadroughts (*38*, *39*). In addition, we use the WNATA in conjunction with preexisting gridded reconstructions of warm (May to July) and cool (December to February) season precipitation (*40*) to evaluate spatiotemporal trends in the occurrence of compound hot and dry conditions over the past several centuries.

## RESULTS

### Reconstructed summer temperatures across WNA since 1553 CE

**Fig. 1.**
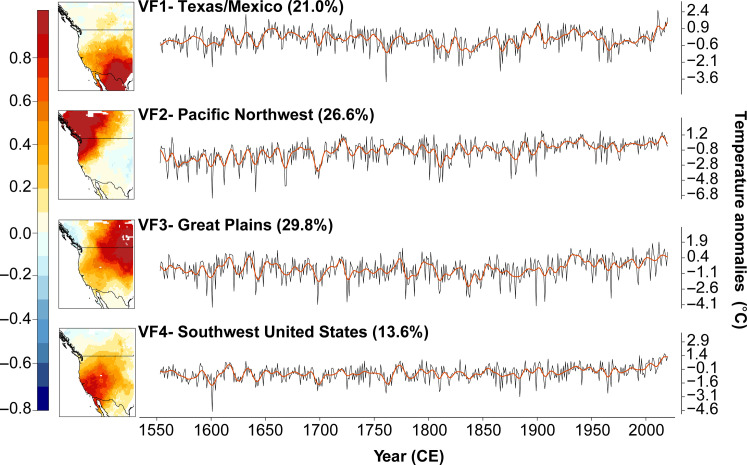
Subregional expression of reconstructed temperatures across WNA since 1553 CE. (**Left**) First four varimax-rotated EOF factor scores (ranging from −1.0 to +1.0) are mapped and labeled with the variance explained by each factor*.* (**Right**) Annual (thin black line) and 10-year low pass–filtered (thick red lines) reconstruction time series of JJA maximum temperatures for four major regions of WNA, spanning the period 1553 to 2020 CE. Anomalies are relative to the 1951 to 1980 CE mean. The four regional time series are calculated using the rotated varimax factor loadings over the period 1901 to 2000 CE.

We use an extensive network of tree-ring chronologies (fig. S1) as the predictors for a spatial reconstruction of summer maximum temperatures extending back to at least 1553 CE. Density and BI records comprising the WNATA network exhibit significant (*P* < 0.01) positive relationships with warm season (April to September) monthly average maximum temperatures, with the strongest average monthly temperature response occurring in August (fig. S2). All WNATA tree-ring chronologies are representative of densiometric tree growth (see the Supplementary Materials), thus, it is expected that the maximum temperature response coincides with the seasonal timing of secondary cell wall thickening (*41*), characteristic of temperate North American conifers. Our reconstructions explain at least 40% of the variance in both the calibration and verification periods across most WNA (fig. S3). Positive validation statistics (*42*) indicate adequate model skill and temporal stability across much of the region. The spatial patterns of reconstructed temperature variability are further corroborated by the similar patterns in the leading empirical orthogonal functions (EOFs) in both the reconstructed and the instrumental data (fig. S4), explaining 90.8 and 72.5% of their total variability, respectively. We applied a varimax rotation to the first four EOFs of the WNATA and used the factor loadings from these rotations to determine the geographic bounds of the four subregional, spatially averaged temperature reconstructions: VF1-Texas/Mexico, VF2-Pacific Northwest, VF3-Great Plains, and VF4-southwest United States (Fig. 1). Pearson’s correlation values between the four-leading varimax-rotated EOF time series based on the reconstruction and the instrumental data (*r* = 0.42, 0.72, 0.67, and 0.66; 1901 to 2000 CE; fig. S5) further reflect the overall strong skill of the WNATA for capturing the spatial nature of temperature variability at 0.5° resolution. However, the comparatively low skill of the WNATA in the Texas/Mexico subregion reflects the limited temperature-sensitive tree-ring collections in the WNATA network in this area (fig. S1), and results pertaining to this part of WNA should be interpreted with caution. The subregional-average reconstructions reflect multidecadal trends in temperature variability, most notably documenting a steady warming trend spanning ~1960 to present. Reconstructed summer temperatures also reflect significant (*P* < 0.01) seasonal cooling following major volcanic eruptions (fig. S6). Subregional WNATA reconstructions are highly correlated and exhibit similar multidecadal patterns of temperature variability with preexisting reconstructions back to 1600 CE based solely on tree-ring maximum latewood density (MXD) (fig. S7) (*43*). However, the WNATA dataset allows for the examination of temperature for several decades to several centuries further back in time, and the reconstruction models calibrate over an additional 20 years forward in time. The temporal extension back to 1553 CE allows for direct and independent comparisons of maximum temperature and drought through recent times including during the late 16th-century megadrought.

### Comparing modern summer *T*_max_-PDSI relationships to those in the past

**Fig. 2.**
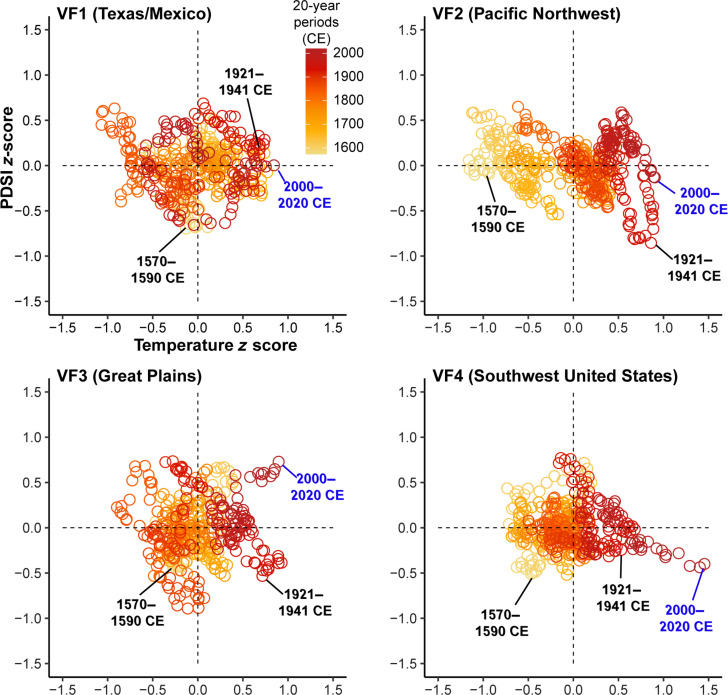
Bivariate distribution of 20-year moving averages of summer maximum temperature and PDSI across four subregions of WNA. Twenty-year moving averages of regionalized summer temperature *z* scores from the WNATA and PDSI values from the LBDA are relative to the full period (1553 to 2020 CE). Averages are calculated using a 20-year backward moving window beginning in 2020 CE. For each subregion, the average *T*_max_ and PDSI anomaly for the 2000 to 2020 CE period is annotated in blue. Select 20-year periods capturing the Dust Bowl (e.g., 1921 to 1941 CE) and 16th-century megadroughts (e.g., 1570 to 1590 CE) are annotated in black.

We first compare the WNATA temperature reconstructions with collocated and data-independent dendroclimatic reconstruction estimates of JJA Palmer’s Drought Severity Index (JJA PDSI) (*44*) from the Living Blended Drought Atlas (LBDA) (*37*). Over the shared period (1553 to 2020 CE), the grid point reconstructions of JJA *T*_max_ and PDSI are significantly (*P* < 0.01) negatively correlated (fig. S8). Across WNA, 50-year Pearson’s correlations between the WNATA and LBDA demonstrate that the negative relationships between *T*_max_ and PDSI are the most spatially contiguous in recent decades (fig. S9). To evaluate multidecadal *T*_max_-PDSI relationships at the subregional scale, we calculated spatially averaged PDSI reconstructions from the LBDA over the same four areas used to produce the subregional WNATA temperature reconstructions. Bivariate distributions of 20-year moving averages from the subregional *T*_max_ and PDSI reconstructions indicate that the most recent decades are among the warmest and driest multidecadal periods across WNA (Fig. 2). The two decades from 2000 to 2020 CE is the warmest period of the past five centuries across the southwestern United States, Pacific Northwest, and Texas/Mexico and ranks second behind the 1929 to 1949 CE “Dust Bowl” (*37*) average for the Great Plains. Both the Great Plains and Pacific Northwest subregional averages indicate that periods encompassing the Dust Bowl (e.g., 1921 to 1941 and 1929 to 1949 CE) exhibit similar *T*_max_ averages relative to modern times, but they reflect more severe 20-year average soil moisture deficits. The 2000 to 2020 CE average PDSI value for the southwestern United States rivals those of the 20-year periods encompassing the 16th-century megadrought (e.g., 1570 to 1590 CE) as being the driest, and the *T*_max_ averages for the most recent decades far surpass those experienced during the late 16th century.

Over the past five centuries, megadrought periods show substantial spatiotemporal variability in the relationship between summer *T*_max_ and PDSI across WNA (fig. S10). However, despite the varying durations and spatial footprints of WNA megadroughts, grid point locations where the average PDSI anomalies are most severe during these times also experience some of the highest average temperature anomalies. Evaluating WNA as a whole, summer temperatures recorded during the Dust Bowl (1932 to 1939 CE) (*23*) and the modern (2000 to 2020 CE) (*10*) megadrought are, on average, at least 0.6°C warmer than during any other historical WNA megadrought (fig. S11). Notably, the late 16th century and modern periods are analogous in terms of duration, spatial footprint, and average soil moisture anomaly; however, the former is not characterized by exceptionally warm summer maximum temperatures. This pattern is especially apparent in the Pacific Northwest region, where the regional expression of colder temperatures during the Little Ice Age is particularly pronounced (*45*).

**Fig. 3.**
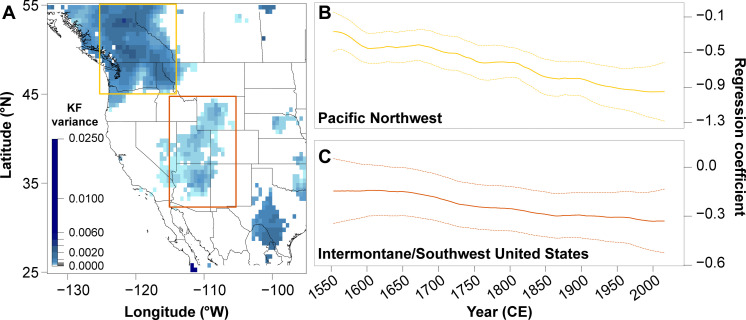
Dynamic regression modeling using a KF reveals a strengthening relationship between reconstructed maximum temperature and Palmer’s modified Drought Severity Index across WNA over the period 1553 to 2020 CE. (**A**) KF variance resulting from dynamic regression modeling using a KF filter between the LBDA and WNATA time series for each shared grid point. Only variances >0 that also meet the Akaike information criteria are shown in blue. The two selected regions of high variance are denoted with colored boxes. For each of the two identified regions, the regional average of the KF traces (regression coefficients; solid, colored lines) are plotted in (**B**) for the Pacific Northwest and (**C**) for the Intermontane/Southwest United States with 95% confidence intervals (dashed, colored lines), where a 30-year high-pass filter was applied to the LBDA and WNATA before regression modeling.

To further evaluate relationships between summer *T*_max_ and PDSI over the past several centuries, we apply a dynamic regression model using a Kalman filter (KF) (*46*) between the WNATA and LBDA at each shared grid point (see Materials and Methods). Locations exhibiting the highest KF variance are interpreted as places where the greatest change in the coupled temperature-drought relationship has occurred; the KF variance is highest over the Pacific Northwest and the intermontane/southwest United States (Fig. 3). For both regions, averaged KF coefficients trend increasingly negative, indicating a strengthening of the negative relationship between PDSI and *T*_max_ at high-frequency time intervals. The decades spanning the modern megadrought exhibit the strongest negative relationships between summer maximum temperatures and soil moisture compared to any other historical period of megadrought conditions experienced across these regions of WNA. Although grid points where the KF variance is highest often correspond with some of the most data-rich portions of the WNATA network, we find no statistical evidence linking the KF variance to either the relative skill of the reconstruction or the number of predictors retained for each WNATA grid point reconstruction (fig. S12).

### Quantifying the historical prevalence of co-occurring warmth and precipitation deficits

**Fig. 4.**
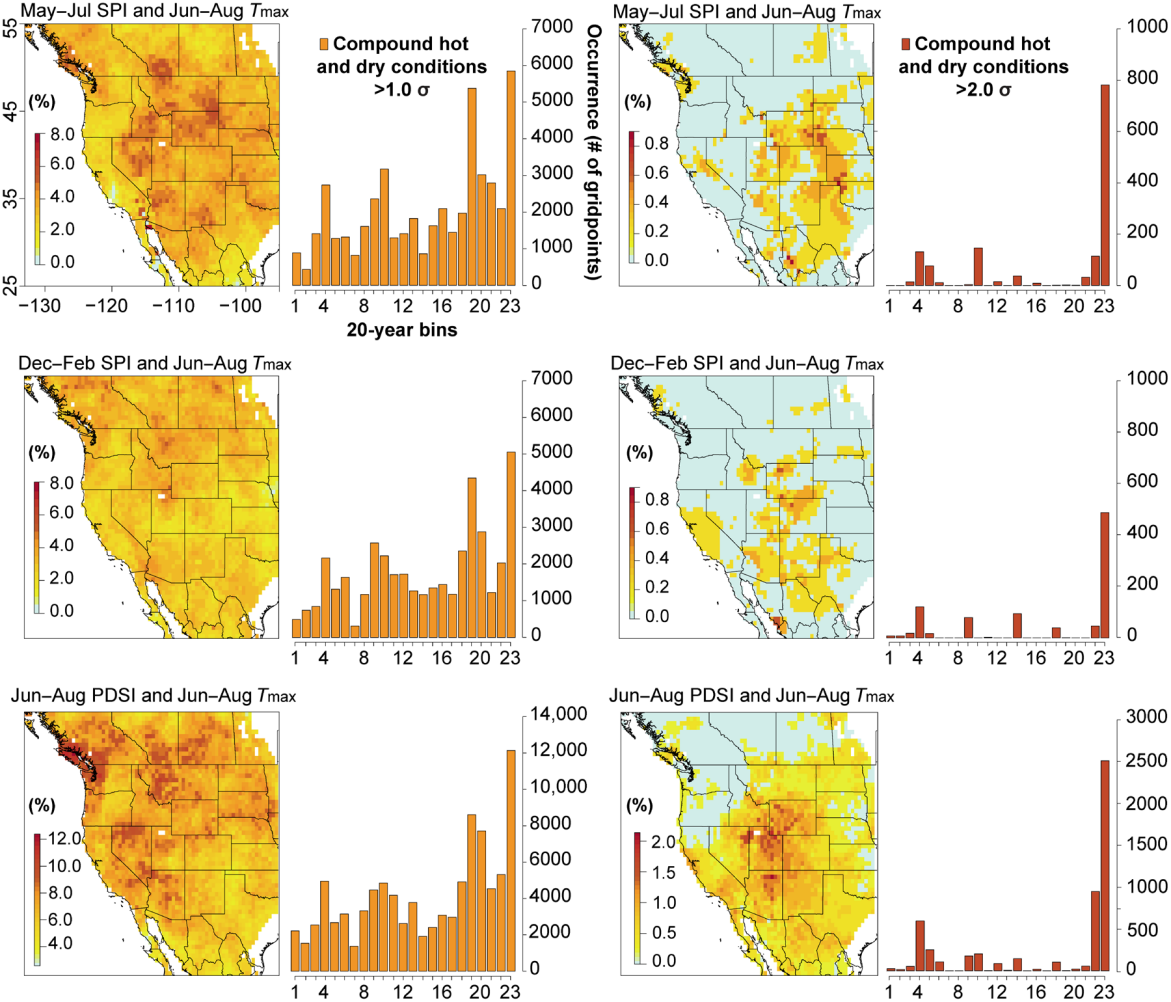
Historical occurrence of compound hot drought (hot and dry conditions) across WNA since 1553 CE. The fractional probability of occurrence of compound hot and dry conditions is mapped, where in any given year over the period 1553 to 2016 CE, each grid point location experiences concurrent warm season (May to July; **top row**), cool season (December to February; **middle row**), standardized precipitation index deficits, or warm season (JJA; **bottom row**) PDSI deficits and positive summer maximum temperature anomalies exceeding 1.0 σ (**left column**) and 2.0 σ (**right column**). Counts of occurrence at the grid point level of compound warm and dry summer conditions are plotted in 20-year bins to the right of each map. For bar plots, year corresponding to bin numbers 1 to 23 are: 1) 1557 to 1576, 2) 1577 to 1596, 3) 1597 to 1616, 4) 1617 to 1636, 5) 1637 to 1656, 6) 1657 to 1676, 7) 1677 to 1696, 8) 1697 to 1716, 9) 1717 to 1736, 10) 1737 to 1756, 11) 1757 to 1776, 12) 1777 to 1796, 13) 1797 to 1816, 14) 1817 to 1836, 15) 1837 to 1856, 16) 1857 to 1876, 17) 1877 to 1896, 18) 1897 to 1916, 19) 1917 to 1936, 20) 1937 to 1956, 21) 1957 to 1976, 22) 1977 to 1996 and 23) 1997 to 2016 CE.

In addition to comparing the WNATA with the LBDA, we compare the WNATA to both the May to July (warm season) and December to February (cool season) Standardized Precipitation Indices (SPI) from the North American Seasonal Precipitation Atlas (NASPA) (*40*) for the evaluation of compound warm and dry conditions back through time. The dominant mode of total annual moisture delivery is not ubiquitous across WNA. Evaluating both warm and cool season SPI, in addition to summer PDSI, thus potentially allows for a more complete understanding of hot drought occurrence across varying seasonal precipitation regimes. Over the past five centuries, moderate hot drought characterized as above-average warm season temperatures concurrent with either warm or cool season precipitation deficits >1.0 σ is historically most prevalent across the intermountain/southwest United States and the Great Plains regions (Fig. 4). When considering cool season precipitation and PDSI deficits, the Pacific Northwest region shows some of the highest frequencies of concurrent warm and dry conditions >1.0 σ. The spatial footprint of compound warm and dry events, denoted by the cumulative number of grid point locations experiencing concurrent warm and dry conditions, increases sharply in the 20th century compared to the prior ~480 years. This 20th-century increase is present when examining both PDSI and the seasonal precipitation deficits. The 20-year period characterized by the most widespread concurrent warmth and dryness occurs in the most recent decades, with the second-ranking period encompassing the Dust Bowl. We find that the Great Plains region is historically most prone to experiencing anomalously warm and dry summers >2.0 σ, while the central/southern United States Rocky Mountains and much of California show the highest historical prevalence of concurrent warm summer temperatures and low winter precipitation. Once more, the spatial footprint of severe hot drought over the past two decades far exceeds that of any other period since at least the middle of the 16th century.

## DISCUSSION

Improved spatial reconstructions of past summer temperature from independent tree-ring data collocated with existing hydroclimate reconstructions is essential for the independent evaluation of temperature-drought relationships through time (*47*). Our reconstruction now permits important findings regarding the influence of summer temperatures on the development of notable past droughts in WNA. For example, the late 16th-century megadrought may be comparable to the modern southwestern U.S. megadrought in terms of severity (cumulative soil moisture deficit), but our results indicate that the earlier event was not a hot drought analogous to the early 21st-century megadrought. The most recent drought is exacerbated by high temperatures (*10*, *24*), which substantially exceed those during the late 16th century. Although it has been long-established that precipitation deficits during WNA megadroughts over the past millennium are driven by sea surface temperature (SST) variability, our results here require that we examine another potential driver that may help explain the differences between modern and 16th-century drought conditions: The link between increased evaporative demand and anthropogenically accelerated temperature increases.

As expected, summer *T*_max_-PDSI relationships across WNA are consistently negative over the past several centuries, with the most recent decades reflecting the strongest negative coupling. The documented strengthening of the association between summer *T*_max_ and PDSI over the past century is consistent with increasing aridity trends due to anthropogenic warming across much of the region (*32*, *48*), and we ultimately speculate that this pattern reflects a combination of the feedback pathways characterizing temperature-soil moisture interactions. Negative *T*_max_ and PDSI relationships are not unexpected, given that circulation anomalies that induce subsidence will simultaneously suppress precipitation, drive adiabatic warming, and potentially reduce cloud cover and enhance solar absorption at the surface. However, the strengthening of the temperature-drought relationships through time likely reflects altered land-surface interactions due to anthropogenic climate change, which perturbs these relationships through positive feedback interactions and amplified evapotranspiration (*23*, *37*). Across WNA, amplified evapotranspiration exacerbates surface drying in areas where precipitation is already reduced and can also lead to drought conditions in places that would otherwise experience minimal drying from precipitation trends alone (*49*). Anthropogenic alterations of land-surface feedbacks have been previously linked to the amplification of warmth and drying across the Great Plains region of WNA during the Dust Bowl drought (*23*). Furthermore, multidecadal drying exacerbated by high temperatures may further alter surface energy balance in ways that lead to additional warming. This type of temperature-soil moisture interaction is particularly relevant for the occurrence of extreme hot temperatures and heat waves. For example, Bartusek *et al.* (*50*) argue that multidecadal drying experienced across the Pacific Northwest is contributing to the increased prevalence of hot and dry summers. In addition to the influence of anthropogenic warming, the dynamic relationship between *T*_max_ and PDSI through time is likely influenced by competing hydroclimatic controls on regional PDSI values. These controls, such as whether the dominant control on summer moisture variability is antecedent winter snowpack or the North American Monsoon, vary across WNA. For example, unlike in the intermontane/southwest U.S. region, dynamic regression modeling without prefiltering of the WNATA or LBDA for the Pacific Northwest indicates that the negative KF coefficients do not consistently strengthen through time (fig. S13). This likely reflects the influence of multidecadal snowpack variability across the region (*51**–**53*). Nevertheless, our discovery of the increased association between *T*_max_ and PDSI over the past several centuries corroborates evidence of historically energy-limited regions of North America becoming more moisture-limited because of increases in surface net radiation and higher temperatures over the 20th century (*54*, *55*).

The frequency of moderate and severe hot drought across WNA over the past century has no precedent since 1553 CE. The increasing prevalence of hot drought over the 20th and 21st centuries has important implications for future regional climate change adaptation strategies and for water resource management, particularly in the most historically drought-prone regions. Our analysis identifies the Great Plains as the region most historically prone to experiencing hot drought in terms of above-average warm summers and reduced summer precipitation. This region also contains the Ogallala aquifer, one of the world’s largest aquifers, which supports nearly 25% of all irrigated agricultural ground water for the United States (*56*). Concurrent increases in temperature and decreases in summer precipitation in this region during the growing season may require greater water withdrawals to support agriculture, thereby increasing the risk of accelerated aquifer depletion (*57*). Similarly, portions of the Colorado River basin also are highlighted as historically prone to severe hot drought. The increased prevalence of hot drought in recent decades has already been linked to reduced river flow across many regions of the United States (*48*), including numerous large and growing urban centers that critically depend on a tenuous supply of freshwater. Of the 19% reduction in annual flow of the Colorado River during 2000 to 2014 relative to a 1906 to 1999 baseline, it is estimated that about one-third was due to unprecedented temperatures (*58*). While the future of precipitation in the region remains uncertain, projections of increasing temperatures pose substantial risk for intensifying drought conditions and increasing water insecurity (*58**–**60*) for these economically important, population-dense, and historically active megadrought regions.

Collectively, the paleoclimate records analyzed here corroborate conclusions from a growing body of literature that examine the instrumental record and provide evidence that anthropogenic activity has already led to an increase in both the frequency and severity of concurrent warm and dry summer conditions across parts of the WNA (*61*). Our results demonstrate the imprint of the anthropogenic warming trend and the amplification of warm summer temperatures across WNA over the past century. Furthermore, for much of WNA, the bivariate relationship between summer maximum temperatures and PDSI is stronger during the modern megadrought than during any other historical period of megadrought conditions. Our results also corroborate previous findings from Williams *et al.* (*10*), which show substantial contributions (58%) of anthropogenic trends in temperature and relative humidity to the 2000 to 2018 average soil-moisture anomaly over southwestern North America. The increased association between rising temperatures and declining soil moisture over the past few decades coincides with an increased frequency of hot drought across WNA, thereby providing evidence in support of the argument that bivariate interactions between temperature and soil moisture are at least one of the driving forces exacerbating the prevalence of hot drought across WNA. However, it is still less clear as to whether the circulation anomalies associated with high pressure and subsidence that drive droughts are themselves being intensified or made more likely by climate change or perhaps themselves responding to changing land-surface conditions. Nevertheless, Seager *et al.* (*62*) identified a multidecadal trend toward a cool season ridge at the west coast of North America that would tend to suppress precipitation. In this regard, our results showing an increase in the occurrence of compound summer warmth and declined winter precipitation in the 21st century also align with results from Seager *et al.* (*63*), which suggest that 21st-century summer megadrought conditions over the southwestern portion of WNA are closely linked to winter precipitation deficits driven by decadal variations in SSTs. It is evident that more work is needed to examine whether regions across WNA are undergoing both a transition in land-atmosphere coupling that favors hot droughts and a change in circulation that is suppressing precipitation and drying soils and/or inducing adiabatic warming. Nevertheless, our comparisons of modern conditions to historical analogs suggest that climate change has already altered the drivers of drought severity in WNA over the past ~500 years. As model simulations show that climate change is projected to substantially increase the severity and occurrence of compound drought and heatwaves across many regions of the world by the end of the 21st century (*63*, *64*), it is clear that anthropogenic drying has only just begun (*10*). We advocate for the continued synthesis of paleoclimate data elsewhere in the world to achieve a more complete understanding of regional- to hemispheric-scale drivers of extreme compound climatic extremes.

## MATERIALS AND METHODS

### Climate data

The target field for the WNATA temperature reconstruction is 0.5° CRU TS 4.06 (land) maximum temperature (*64*), averaged over JJA *T*_max_ for the period spanning 1901 to 2020. To evaluate spatial patterns and relationships of temperature and drought over the observational period, we use the global CRU TS 4.05 PDSI data, spanning 1901 to 2020 (*65*, *66*). The WNATA reconstructions are augmented with CRU instrumental JJA *T*_max_ over the period 2001 to 2020 CE (see Supplementary Text). Similarly, the LBDA reconstructions are augmented with instrumental PDSI data spanning 2006 to 2020 CE.

### Tree-ring data

This study uses a network of tree-ring chronologies comprising a combination of preexisting, previously published BI (*67*) and density data (e.g., MXD) (*43*, *68*) from the International Tree Ring Databank, as well as new BI chronologies specifically developed for inclusion in this study (Fig. 1; see Supplementary Text). The network of tree-ring chronologies used to create the WNATA is completely independent of the tree-ring network used to create the NASPA or the LBDA.

All WNATA tree-ring chronologies are standardized to preserve as much low-frequency temperature variability as possible, especially that which relates to 20th-century warming. Assuming that the BI and density parameters rarely increase with age due to biological or geometrical factors, all chronologies are initially detrended following the guidelines of Wilson *et al.* (*69*) and Heeter *et al.* (*70*) based on an age-dependent spline (ADS) (*71*) applied within the signal-free (SF) standardization framework (*72*) and modified with nonincreasing end constraints (*69*). Detrended chronologies are then screened against their local 0.5° JJA *T*_max_ data (fig. S2). Using the SF-ADS detrending approach most frequently returns the strongest calibration results between the chronologies and CRU instrumental JJA *T*_max_ data, but some modifications to detrending are performed for a small number of individual sites. Only chronologies exhibiting significant (*P* < 0.01) positive correlations with current year JJA *T*_max_ are retained for inclusion in the WNATA network for reconstruction purposes.

### Reconstructing summer maximum temperatures using tree-ring network

We use a nested ensemble point-by-point regression (EPPR) to reconstruct summer season surface air temperatures. The EPPR method of climate field reconstruction is comprehensively described in Cook *et al.* (*73**–**75*). Unlike previous applications of EPPR that used multiple search radii for locating candidate tree-ring chronologies (*73*, *74*), we use a single search radius of 1200 km for reconstructing each JJA *T*_max_ grid point, with a minimum of four chronologies used for each grid point reconstruction. The 1200-km search radius is based on the correlation decay (*e*-folding) distance used for interpolating single-station temperature data to the 0.5° grid of the target CRU TS 4.06 land *T*_max_ dataset (*64*). In principle, using this search radius should locate only the candidate predictors that are most likely to be physically related to the collocated temperature data at the reconstruction grid point (*75*).

For the first of three total reconstruction nests, we calibrate and validate each grid point reconstruction over the period 1901 to 1980 CE, as this is the common period shared between the instrumental temperature data and all tree-ring predictors in the WNATA network. We use a split calibration/verification approach (*76*), where we calibrate over the period spanning 1941 to 1980 CE and verify on the period spanning 1901 to 1940 CE. We repeat this approach for the remaining two forward nests: 1901 to 1990 CE and 1901 to 2000 CE. The verification period remains constant, but the calibration period varies for each nest (1941 to 1980 CE, 1941 to 1990 CE, and 1941 to 2000 CE). For each reconstruction nest, we apply eight weights to each of the selected tree-ring predictors based on their correlations with temperature, as described in Cook *et al.* (*73*), resulting in a 24-member ensemble. The mean of this ensemble is recalibrated to provide the goodness of fit of each 0.5° grid point reconstruction using two calibration statistics: the coefficient of determination (*R*^2^) and the cross-validation leave-one-out reduction of error statistics. Reconstruction accuracy is determined for the validation period of withheld data using the explained variance (VRSQ), reduction of error (VRE), and coefficient of efficiency (VCE) statistics (*42*, *76*, *77*). Positive VRSQ, VRE, and VCE values indicate different measures of model skill and are expressed in units of fractional explained variance over the validation period, whereas negative values indicate no reconstruction skill (*42*, *77*). After final calibration and validation of the ensemble mean, the final WNATA reconstruction spanning 1553 to 2000 CE is rescaled at each grid point to recover lost variance due to regression. This enables the WNATA reconstructions to be extended to 2001 to 2020 CE with instrumental data. A similar approach is also used to augment the LBDA, which originally ends in 2005 CE.

To further assess reconstruction skill, we use EOF analysis to compare spatial patterns of the leading modes of temperature variability across the region between the reconstructed temperature estimates and the instrumental data over the shared period (1901 to 2000 CE). Eigenvalue traces are calculated from the correlation matrices of each dataset. For both the reconstruction and the instrumental data, we plot the eigenvalue traces with uncertainty expressed as ±2 SEs (*78*) out to the order 20 and map the actual and reconstructed EOF loadings for comparison. For the subset of eigenvalues traces of each dataset that separate from the rest, we apply a varimax rotation to more distinctly identify subregional patterns of temperature variability. Using the *n* number of subregions identified by the North criterion, we calculate an average temperature reconstruction for each subregion based on factor loading values ≥0.60. We then compare our WNATA regional temperature reconstructions to the WNA regional temperature reconstructions originally developed by Briffa *et al.* (*43*), which were solely developed from tree-ring MXD records.

### Comparing the WNATA reconstruction to existing hydroclimate reconstructions

We compare the WNATA gridded JJA *T*_max_ reconstruction with estimates of JJA PDSI, May to July SPI, and December to February SPI back through time using the data-independent LBDA and the NASPA, respectively. The LBDA is described by Cook *et al.* (*37*), whereas the NASPA is described by Stahle *et al.* (*44*). Both of these gridded datasets are two of the most current, spatially contiguous, and finest-resolution seasonal reconstructions of hydroclimate fields currently available for North America. While the NASPA and LBDA have substantial tree-ring data overlap, the WNATA is data independent of both of these datasets. The original spatial extent of the WNATA initially encompassed 4080 grid points (encompassing 25° to 60.0°N, 135° to 95.0°W). To ensure complete spatiotemporal overlap between all datasets, this larger grid was reduced to the same 3029 grid points comprising the LBDA and NASPA over the area 25° to 55°N, 135° to 95°W and that extend back to at least 1553 CE. We use 1553 CE as the cutoff year because the majority (95%) of the original set of WNATA grid points extends back to this year; this cutoff also allows for the analysis of the late 16th-century megadrought. For each WNATA and LBDA grid point reconstruction, temperature and PDSI estimates are converted to anomalies relative to the full period (1553 to 2020 CE) average. We first use a bivariate analysis between 20-year moving averages from subregional reconstructions of the WNATA and LBDA to evaluate multidecadal periods of concurrent warm and dry periods. To further evaluate summer temperature-drought relationships back through time, we focus on several known WNA drought periods that are well-documented by the observational or paleoclimate records: the 21st century drought (2000 to 2020 CE), mid-20th century drought (1948 to 1957 CE), the Dust Bowl drought (1932 to 1939 CE), the American Civil War drought (1856 to 1865 CE), the Puebloan drought (1666 to 1671 CE), and the late-16th century drought (1568 to 1591 CE).

To quantify and identify changes in the relationship between JJA *T*_max_ and PDSI over time, we apply the KF (*46*, *79*) to the WNATA and LBDA at each shared grid point. Briefly, the KF estimates the dynamic relationship between two variables in contrast to a constant-coefficient linear model and is done so objectively based on maximum likelihood estimation (*79*). The broader application of the KF in dendroclimatology is comprehensively described in Cook and Johnson (*80*), Jacoby and D’Arrigo (*81*), Cook *et al.* (*74*), and Allen *et al.* (*82*). To identify grid points where the dynamic regression model provides a truly better fit to the data compared to the constant-coefficient model, we use the modified Akaike information criteria (AIC) (*83*), where the AIC of the dynamic model (AIC_t_) must be smaller (i.e., explain more variance) than the AIC value of the constant coefficient model (AIC_c_) by at least two (the penalty term of the AIC to account for additional parameters) (*84*). For each grid point where AIC_t_ − AIC_c_ < 2, we assess the variance associated with the final KF fit. A variance >0 with AIC_t_ − AIC_c_ < 2 indicates that the fitted relationship between the two variables over time has improved at a level larger than expected by chance. We then map grid points where these two criteria were met to identify the most likely subregions of WNA where the largest changes to the temperature-drought relationships occurred since 1553 CE. Once these subregions were identified, we calculate regionally averaged time series for the LBDA and WNATA and reapply the KF to the regionalized time series.

For this study, we use the term “hot drought” to describe average summer conditions where any given grid point location experiences the concurrence of anomalously warm conditions and anomalously low warm-season (JJA) PDSI or low warm-season (May to July) or cool-season (December to February) precipitation (based on SPI). This means that the assessment is valid for regions in the west with Mediterranean-type climates and cool-season precipitation only and regions in the interior with cool- and warm-season precipitation maxima or a single warm-season maximum. To evaluate spatiotemporal patterns of hot drought since 1553 CE, we convert the WNATA and NASPA datasets into *z* scores, relative to the full shared period (1553 to 2016 CE). Then, we sum the frequency of occurrence over the full period where any given year is characterized by WNATA *z* scores > 1 σ and NASPA *z* scores < −1 σ. We repeat this method using the criteria of the WNATA showing *z*-score values > 2 σ and the NASPA showing values < −2 σ. For both the 1 and 2 σ criteria, we identify regions that were historically most prone to experiencing compound hot and dry summer conditions by calculating and mapping the relative percent occurrence of hot drought for each grid point. To evaluate changes in the spatial footprint of compound hot and dry conditions across WNA over time, we plot the total occurrence of compound hot and dry conditions over the entire region for both 1 and 2 σ using 20-year bins. For additional comparison, we repeat the aforementioned process using the combination of the WNATA and the LBDA.

## Supplementary Materials

### This PDF file includes:

Supplementary Text Figs. S1 to S13 References
