# Supplementary material for: Increasing prevalence of hot drought across western North America since the 16th century
Source: Sci Adv. 2024 Jan 24;10(4):eadj4289. doi: 10.1126/sciadv.adj4289 (PMC10807802; doi:10.1126/sciadv.adj4289)
Supplement: Supplementary file 1 — Supplementary Text Figs. S1 to S13 References [file sciadv.adj4289_sm.pdf]

Science Advances
AAAS

Supplementary Materials for

Increasing prevalence of hot drought across western North America since the 16th century

Karen E. King *et al.*

Corresponding author: Karen E. King, kking@utk.edu

Sci. Adv. **10**, eadj4289 (2024)
DOI: 10.1126/sciadv.adj4289

This PDF file includes:

Supplementary Text
Figs. S1 to S13
References

Supplementary Text

The ability of tree ring data to capture past temperature variability

Climate proxy records (*e.g.,* tree-rings) provide essential baseline data in the absence of instrumental records and allow for an improved understanding of both modern and pre-instrumental climate variability. Networks empirical tree-ring records, which strongly capture internal variability and can be used to quantify spatiotemporal temperature-precipitation relationships, are important for constraining future modeled scenarios and reducing uncertainties regarding extreme temperature and drought risk for North America (NA). In the past, there were difficulties in developing independent tree-ring reconstructions of temperature for varying regions of NA with which to assess the role of temperature in past drought events, especially at lower latitudes. These difficulties stemmed from increasingly complex hydroclimate-radial growth relationships (*i.e.,* moisture availability exerts a stronger and much more common direct limitation on ring width variability than temperature) (76,85,86). However, the development of additional tree ring metrics that reflect tree-ring wood density rather than radial growth, such as maximum latewood density (MXD) (68) and blue intensity (BI) (67,87), has substantially improved the spatial-temporal coverage of temperature proxy records across WNA (43,45,69,70,88-97).

Although MXD has been used successfully to reconstruct summer monthly temperatures across much of NA (88,89,98,99), the cost-intensive nature of obtaining MXD data is prohibitive to researchers attempting to develop new temperature reconstructions over large spatial extents or to regularly update older MXD sites collected in the 1980s. Thus, reflected visible light-based techniques (*e.g.,* BI methods) have become increasingly popular over the past decade. BI methods, for instance, use the light absorbance properties of wood compounds in the cell walls to obtain a measure of reflectance across portions of an annual growth ring in the blue visible spectral band. Examination of tree-ring minimum latewood BI by McCarroll et al. (67) showed that latewood blue light reflectance exhibited a strong, negative relationship with MXD ($r = -0.95$, $p < 0.01$), thus identifying BI as an important and effective alternative to MXD for developing temperature proxies. Due to the inverse relationship between MXD and latewood blue reflectance (*i.e.,* a dense, dark latewood will show low reflectance), the raw blue light reflectance data are inverted (latewood blue intensity; LWB) to allow for the same interpretations and detrending procedures between the two parameters (94, 100). As such, latewood BI (LWB) chronologies often have strong, positive relationships with summer temperatures that are typically very similar to that of MXD and exhibit less signal contamination from biological memory (physiologically driven autocorrelation) than TRW. Thus, LWB chronologies express autocorrelation that is more similar to that in the instrumental data (101-103). As BI parameters (LWB, earlywood BI; EWB, and delta BI - difference between EWB and LWB; ΔBI) offer cost-effective alternative tree-ring parameters for reconstructing temperature, BI-derived temperature proxies have become important additions to the MXD and TRW temperature proxy network across the Northern Hemisphere (104).

Creation of new tree ring blue intensity (BI) records

While the majority of the tree ring data included in the WNATA network have previously been published (43,45,70,94,96,97) and are now publicly available or available upon request by the original authors, numerous BI chronologies were developed with the primary intent being their incorporation into this project. These new BI records represent a mixture of *i.* previously sampled, loaned collections that were re-evaluated for BI methods and *ii.* recently sampled collections by the authors. For all BI records, prior to actual BI data collection, all radial tree core samples were passively soaked in 99.5% ethanol for 96 hours to remove resin content and then surfaced with 1000 grit sandpaper (45). We then obtained total-ring width measurements using an Epson Expression XL 12000 commercial flatbed scanner in combination with the software CooRecorder (105). Total-ring width measurements were used to visually cross-date each sample. The absolute dating of all tree ring series was statistically validated using the

software, COFECHA (106). After we established absolute dating, we collected BI data for each sample with CooRecorder and following the protocol detailed in Heeter et al. (107). We note that total-ring width chronologies were developed strictly for the purposes of cross-dating the tree ring samples, and they were not included as predictors in the WNATA network.

Reconstructing summer maximum temperatures

To create the WNATA dataset, we employed a nested version of the Ensemble Point-by-Point Regression (EPPR) used to develop the numerous spatial field reconstructions of large area drought, monsoon, and temperature across the Northern Hemisphere (37,40,42,73,74,108,109). EPPR uses a principal components regression to sequentially reconstruct individual grid points of climate over a given field. For this study, a 1,200 km search radius was objectively determined based on the *e*-folding correlation decay distance over the target CRU TS 4.06 JJA T_{max} domain. In principle, using this search radius should locate only the candidate predictors that are the most likely to be physically related to the collocated temperature data at the reconstruction grid point (74). Prior to their inclusion in the WNATA network, all tree ring chronologies were first screened against their local (within 0.5° of site location) CRU TS 4.06 JJA T_{max} data over the common period (1901-1983). We retained chronologies for the initial pool of candidate predictors for reconstruction if they exhibited significant ($p<0.01$) positive Pearson's correlations with the local temperature target. From this initial pool of candidate predictors, tree ring series were then screened for statistical association with the grid point temperature data. The chronologies found within each grid point's 1,200 km search radius had to be positively correlated with current year (t_0) summer temperature over the calibration period ($r > 0$). Here, we used 1941-1980 CE as the common calibration period, and then we included two additional forward nests, calibrating over 1941-1990 CE, then over 1941-2000 CE for a total of three reconstruction nests. As detailed in Cook et al. (74), in requiring $r > 0$, we assume that directly correlated effects of temperature on tree densiometric growth are likely to produce the most reliable expressions of past temperature variability. To account for the potential influence of autocorrelation or trend on the identification of which tree rings were positively correlated with current year temperature, we retained only chronologies exhibiting positive correlations between the calibration period instrumental data and the prewhitened, first-order autoregressive (AR1) version of each chronology. Tree ring predictors were retained for the subsequent reconstructions if they exhibited positive values for three correlation tests: the parametric Pearson product-moment correlation, the non-parametric Spearman rank correlation, and the Robust Pearson correlation. After tree ring chronologies were selected using this process, the original (non-prewhitened/non-AR1) chronologies were used to develop the reconstructions. If at least four chronologies meeting the above criteria could not be found within 1,200 km of a given grid point, the search radius at that grid point was enlarged by 50 km increments until the criteria were met. The we set the minimum acceptable sample size for each chronology to four samples. The ensemble-based approach variably-weights the candidate tree ring chronologies found within the 1,200 km search radius. We used a weighting approach rather than a threshold method (*e.g.,* significant, positive correlation, where $p<0.01$) with the aims of reducing any climatic information that can potentially be lost when employing the threshold method. EPPR utilizes the covariance between the tree ring chronologies and the temperature data in the regression model by weighting each chronology by some power of its correlation with the target temperature variable being reconstructed. The correlation matrix of tree-ring predictors is transformed into a covariance matrix that emphasizes the more heavily weighted (better correlated with climate) tree-ring chronologies. Then, the principal components regression is conducted using the correlation-weighted covariance matrix. The weighting (*wgt*) is related to some power *p* of the positive correlation *r* of each tree-ring chronology with the grid point temperatures being reconstructed ($wgt = r^p$) (74). The r^p weighting applied to each chronology was based on the average *r* of the three correlation tests listed above, where r^p down-weights the more-weakly correlated predictors depending on the choice of *p*, and no down-weighting was applied for the limiting case of $p = 0$ (74). A total of eight powers were applied (p = 0, 0.10, 0.25, 0.50, 0.67, 1.0,

1.5, and 2.0)), thus creating an ensemble of eight reconstructed temperature fields for each of the three nests, which were then averaged to produce an ensemble mean field of 24 total ensemble members.

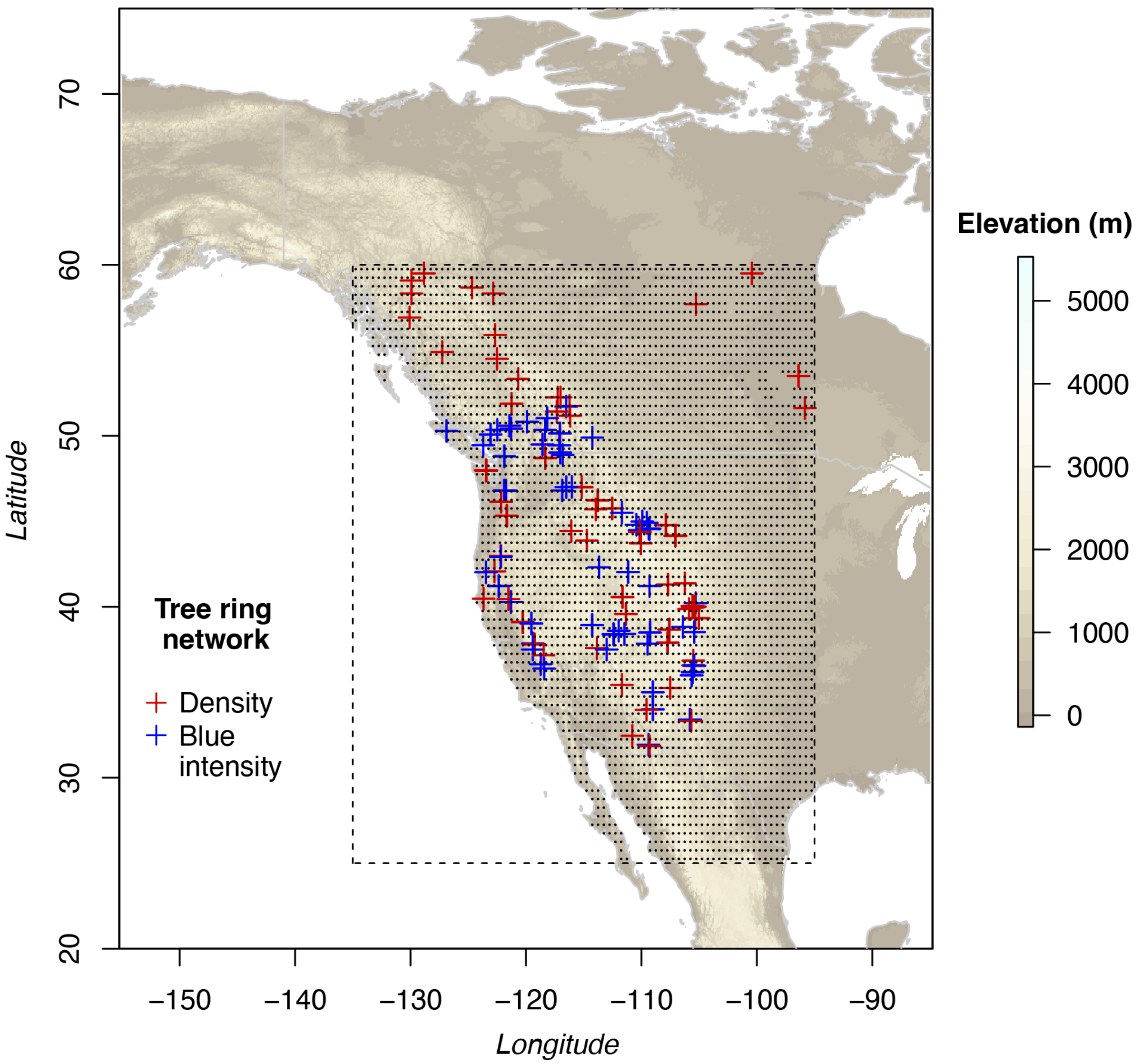

Fig. S1. Reconstruction region and tree-ring network for reconstructing summer maximum temperatures. The spatial distribution of tree-ring predictors, colored by type, are overlaid on a digital elevation model. Density records (n = 145) are in red, and blue intensity records (n= 82) are in blue. The dashed black line delineates the geographic boundary (25-60.0ºN, 135-95.0ºW) from which the target 0.5º CRU TS 4.06 land temperature data was selected for the reconstruction target.

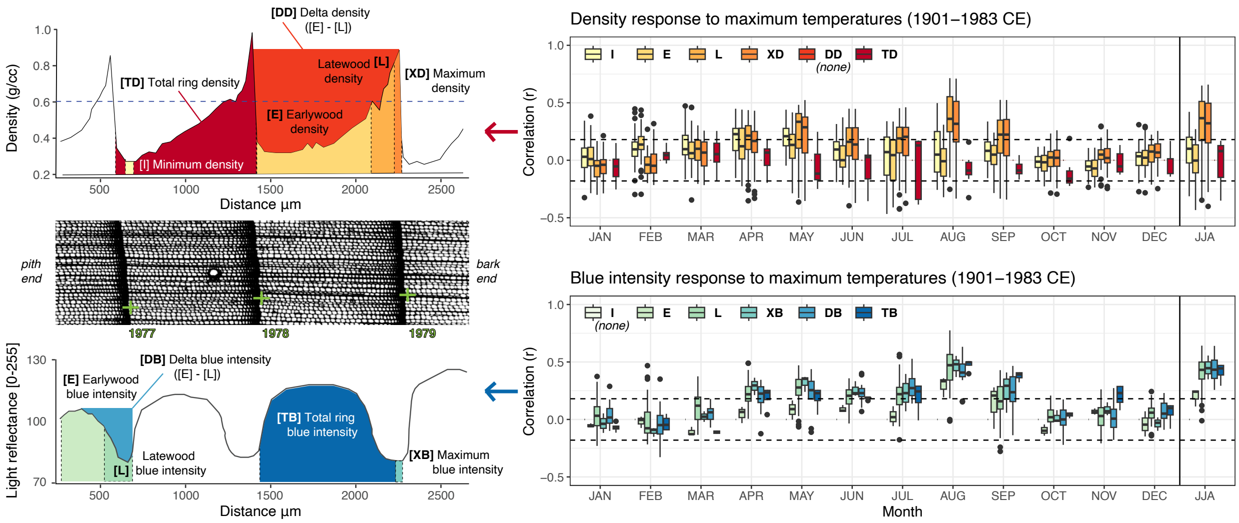

Fig. S2. Conceptual illustration and current-year maximum temperature response of all chronologies initially screened for inclusion in the WNATA network. *(Left)* Demonstrative illustration of a tree-ring density and a blue intensity (BI) profile across several annual spruce tree rings (black and white image, with annual ring boundaries marked with green X's), with the corresponding density and BI parameters comprising the chronologies examined for their inclusion in the WNATA network. Note that BI values are inverted actual blue light reflectance values. *(Right)* Each chronology was screened against their local (within 0.5° of the site location) monthly current-year maximum temperature data CRU TS land 4.06 (spanning 1901- last year of chronology) as well as with summer (June-August; JJA) average maximum temperatures. Chronologies are sorted out by type, which correspond to the different density and BI parameters illustrated in the left-hand side of the figure.

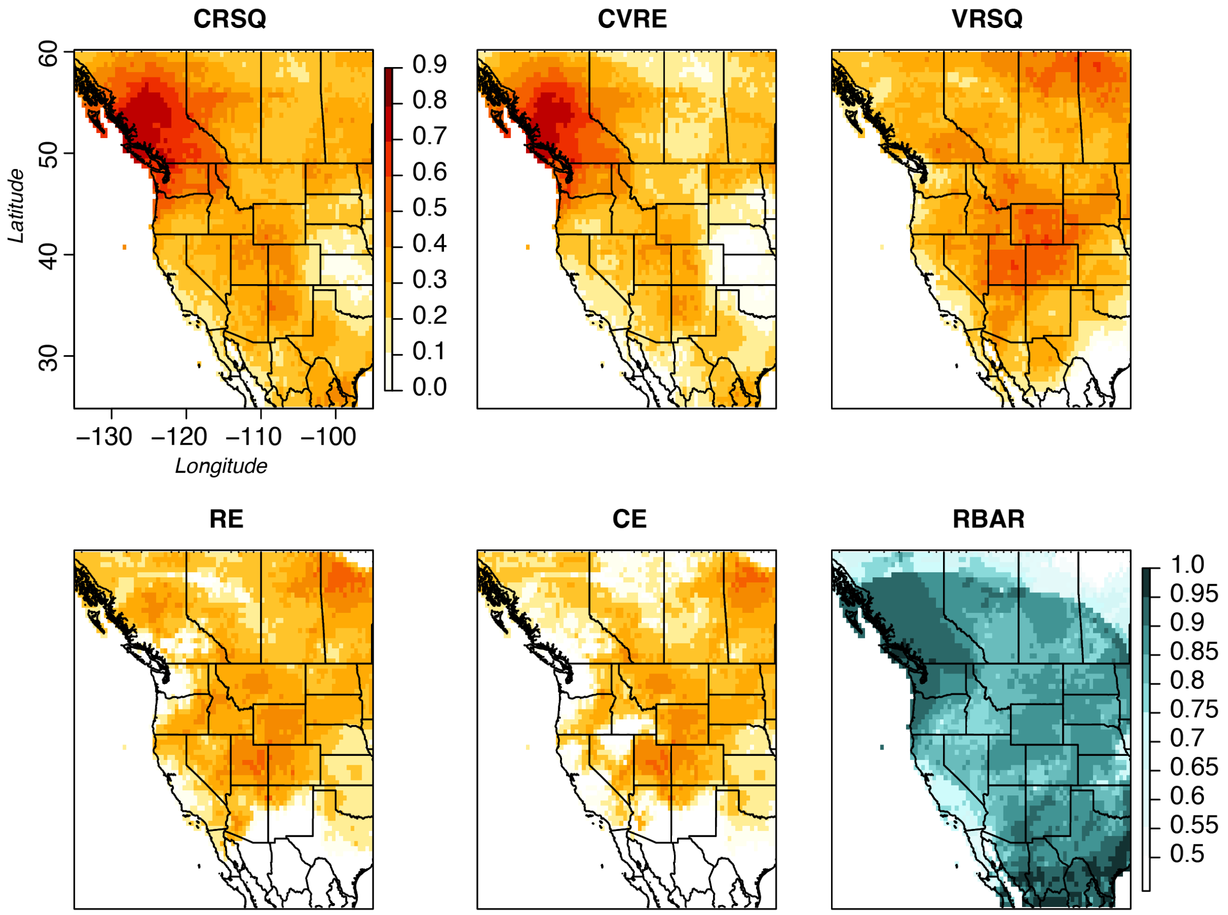

Fig. S3. Calibration/verification statistics for the 0.5º ensemble reconstruction of summer maximum temperatures, based average of 24 EPPR runs. CRSQ calibration-period R^2, CVRE calibration-period leave-one-out cross-validation, VRSQ validation-period square of the Pearson's correlation, VRE validation-period reduction of error, VCE validation-period coefficient of efficiency, RBAR average correlation between the 24 ensemble members.

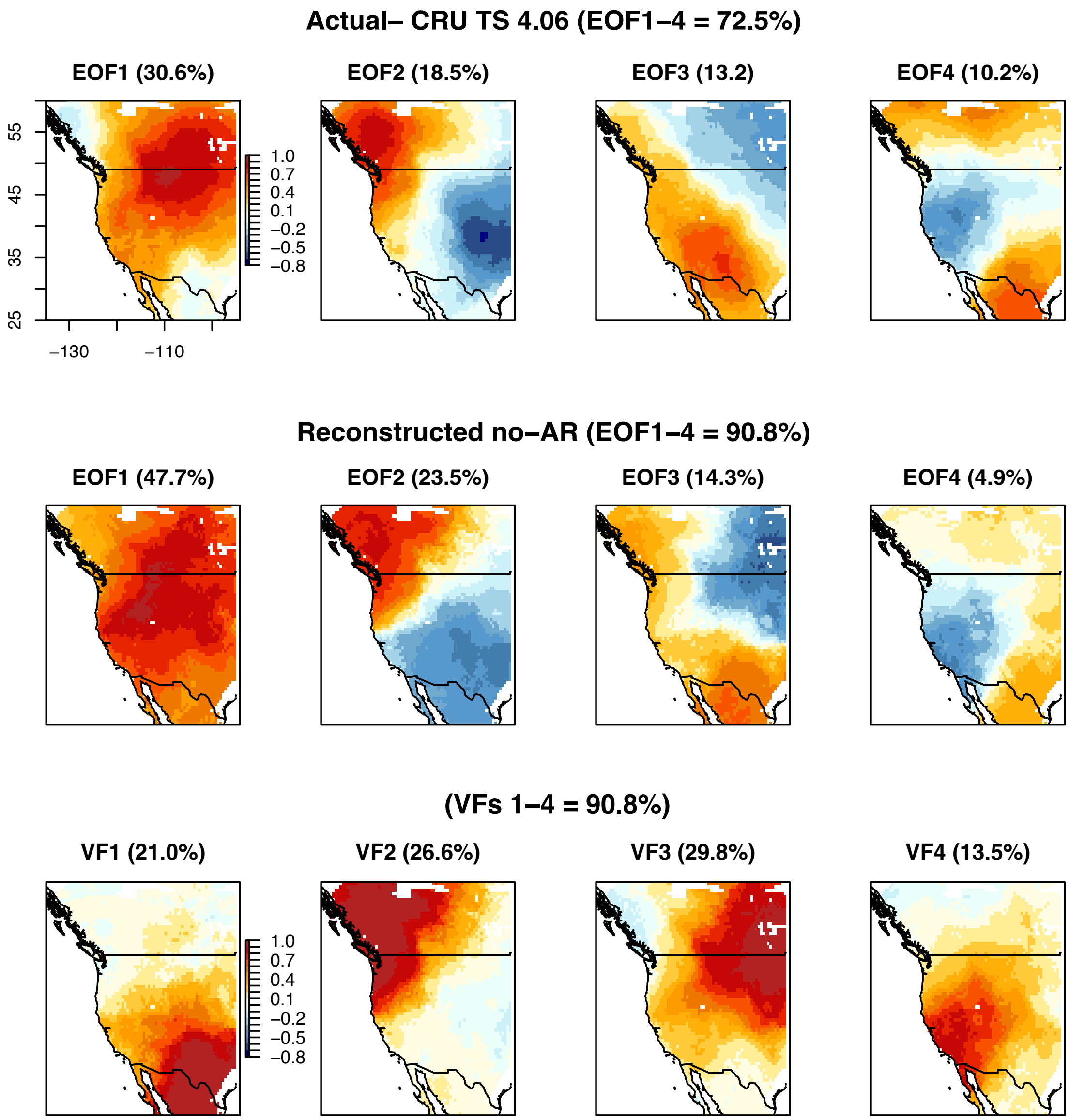

Fig. S4. Mapped unrotated and rotated EOFs 1-4 of reconstructed and instrumental June-August maximum temperatures (JJA T_{max}). Spatial patterns of the four leading unrotated EOFs with explained variance for the 0.5° WNATA JJA T_{max} reconstruction compared against the four leading EOFs for CRU TS 4.06 land JJA T_{max} data, the observational dataset serving as the predictand for the WNATA dataset. Comparisons are made over the common period (1901-2000 CE).

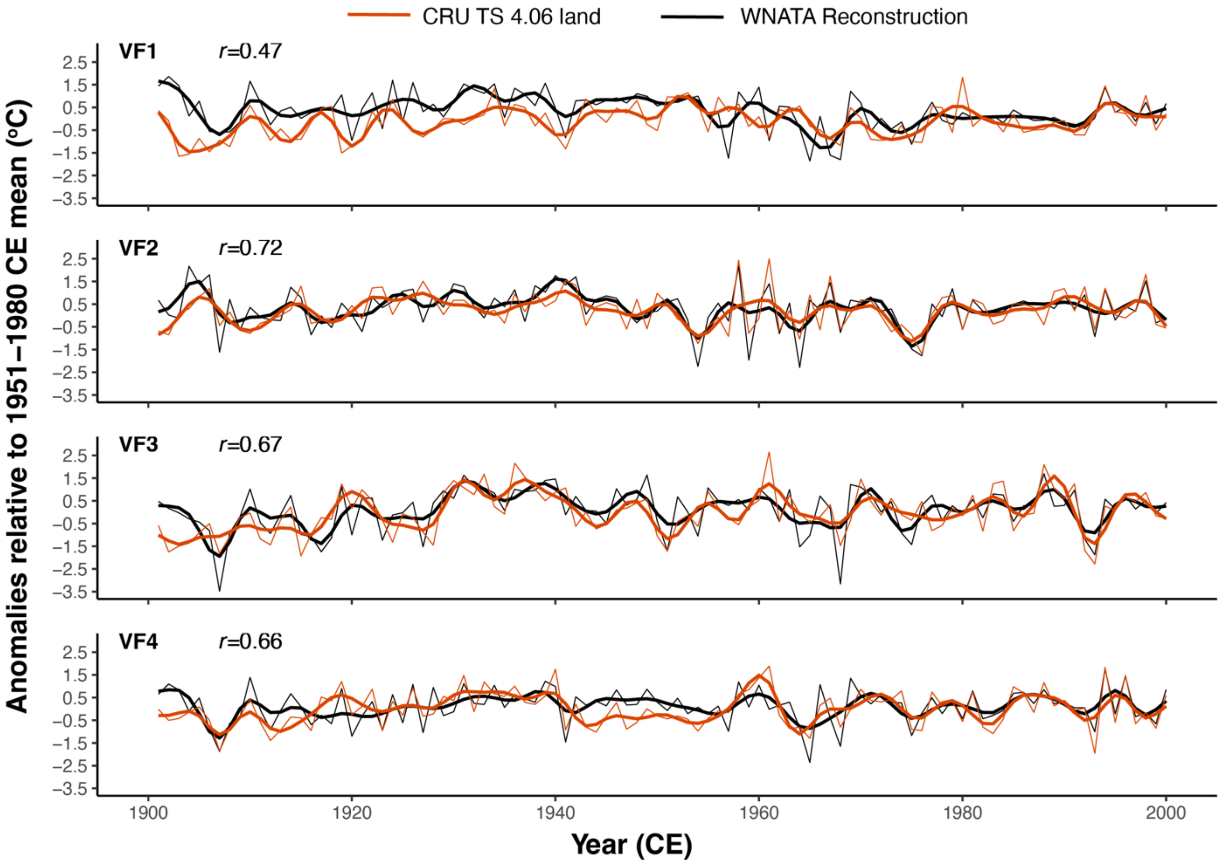

Fig. S5. Comparison of the four leading varimax rotated EOFs based on the instrumental and reconstructed datasets over corresponding regions. Plotted time series of the varimax rotated EOFs 1-4 based on the CRU TS land 4.06 (red line) and WNATA (black line) JJA T_{max} datasets over the period 1901-2000 CE. Temperature anomalies are calculated relative to the 1951-1980 CE mean. Annual timeseries are overlaid with smoothed version of the timeseries using a 5-year low pass filter. Pearson's correlation coefficients between the annual (non-smoothed) instrumental and the reconstructed time series are denoted for each of the respective rotated EOF pairings.

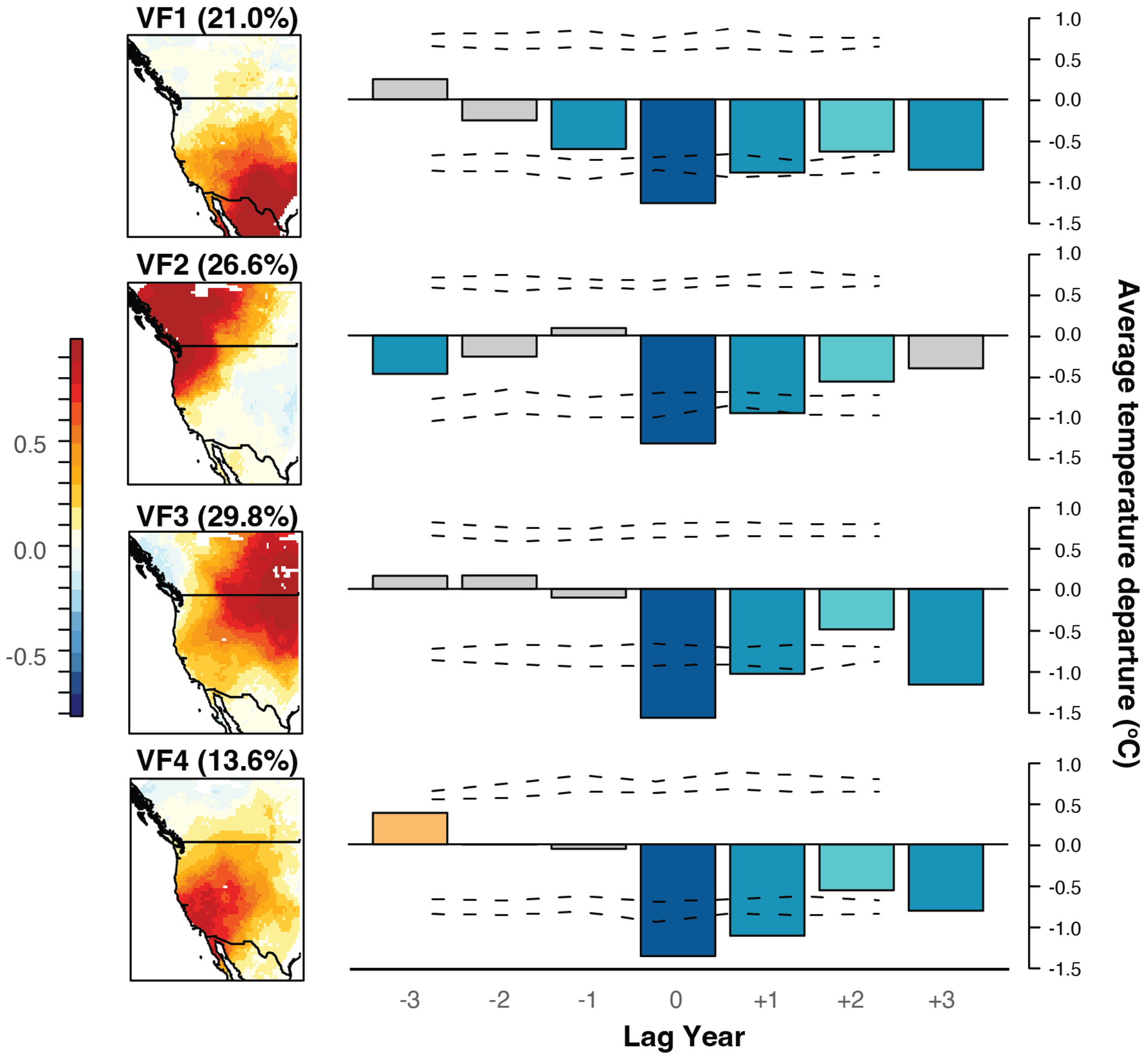

Fig. S6. Reconstructed summer temperatures anomalies and response to major volcanic events across four regions of Western North America since 1553 CE. Superposed epoch analysis between major volcanic events years with a global forcing magnitude equal to or larger than that associated with Krakatoa (1884 CE; n =9; 1595, 1601, 1641, 1695, 1809, 1815, 1832, 1836, and1884 CE) and each of the four regional reconstructions. The four regions are based on the rotated varimax factor loadings over the period 1901-2000 CE Average temperature departures (bars) are shown for +/- 3 years relative to each major volcanic event. Negative, significant temperature departures ($p<0.05$ and $p<0.01$; black dashed lines) are indicated by blue bars.

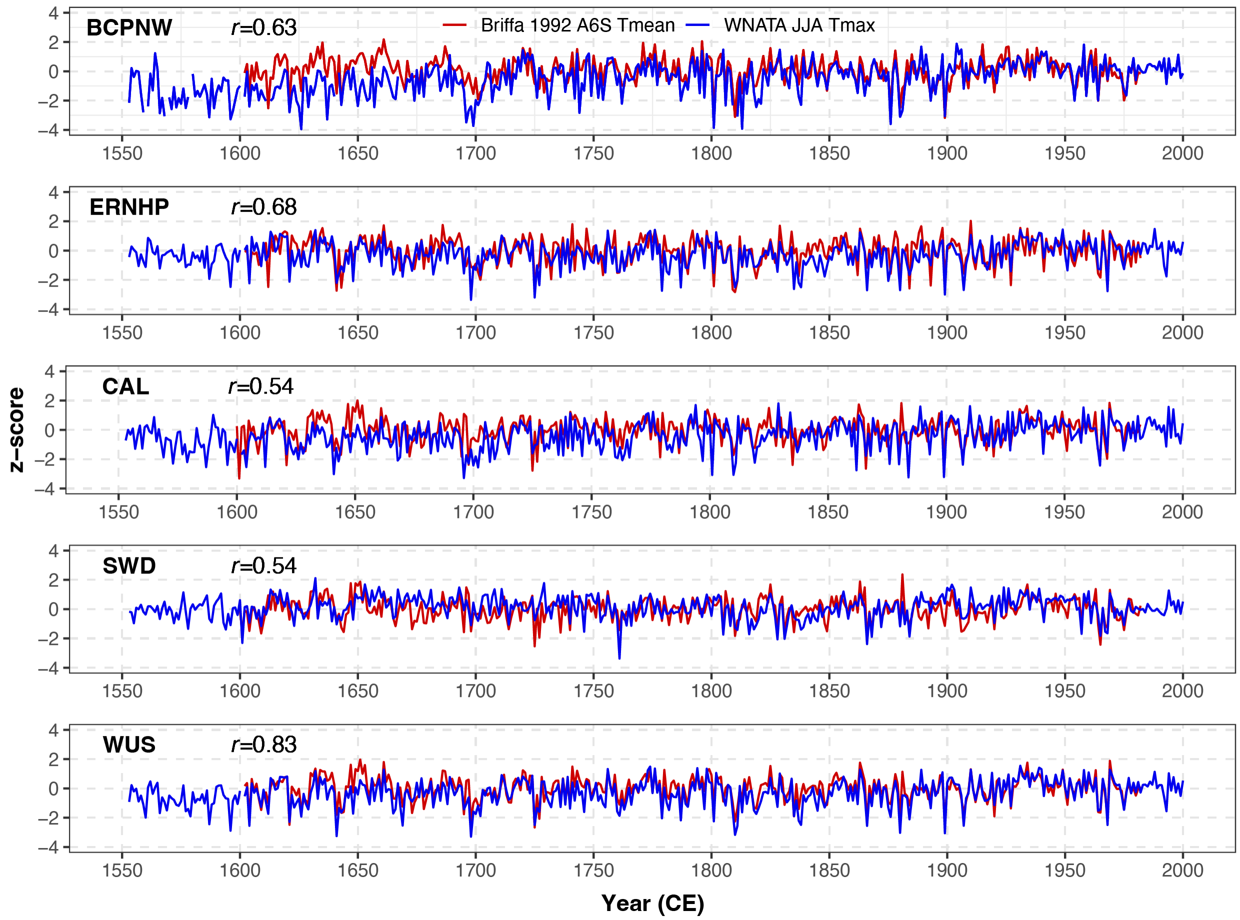

Fig. S7. Comparison of the WNATA June-August maximum temperature and the Briffa et al. (35) April-September mean temperature reconstructions over subregions of western North America. Recalculated regional averages from the WNATA dataset for the five regions detailed in Briffa et al. (35). Regionalized WNATA reconstructions (blue lines) and the Briffa reconstructions (red lines) represented as z-scores, relative to the full shared period (1600-1980 CE). Pearson's correlations (r) between the two reconstructions for each region are also calculated over the full shared period.

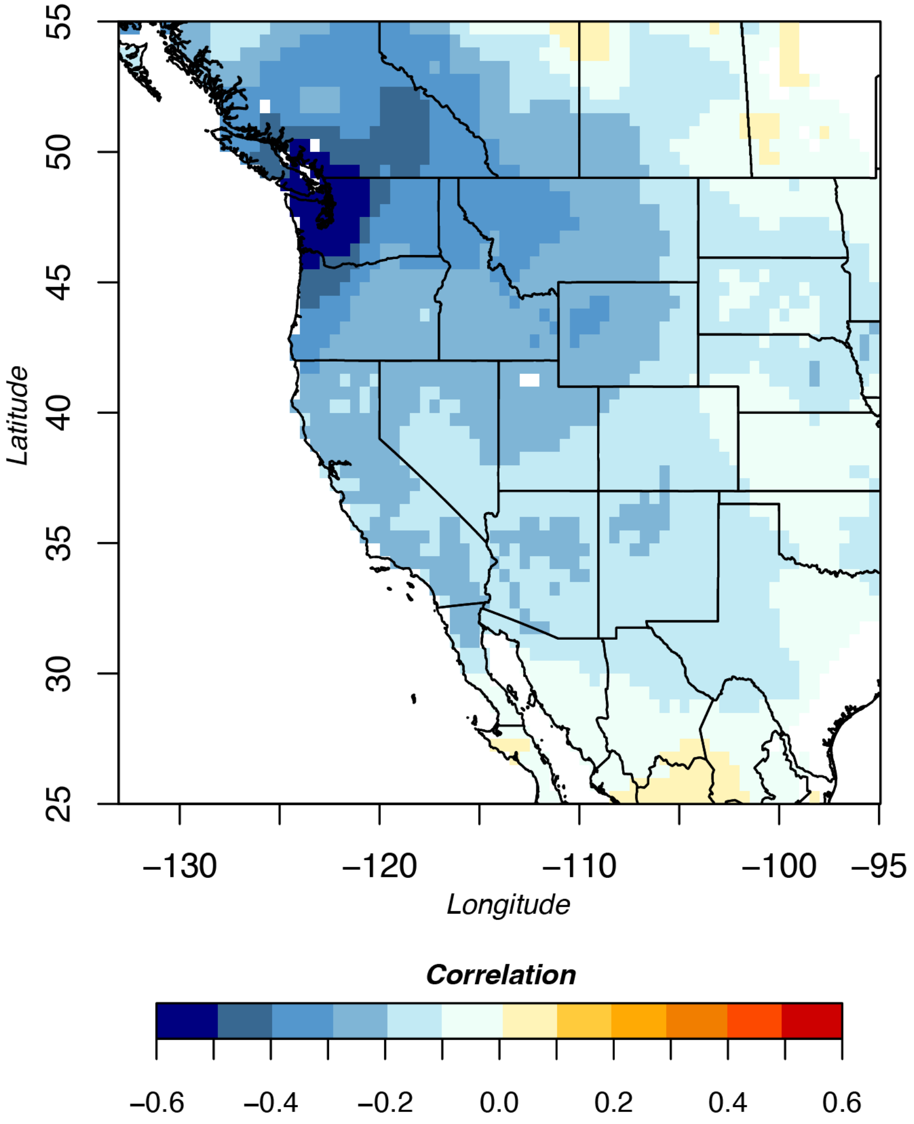

Fig. S8. Mapped correlation values between reconstructed JJA T_{max} and scPDSI (1553-2020 CE). Mapped Pearson's correlation values between summer T_{max} anomalies from the WNATA and scPDSI values from the LBDA over the full period (1553-2020 CE).

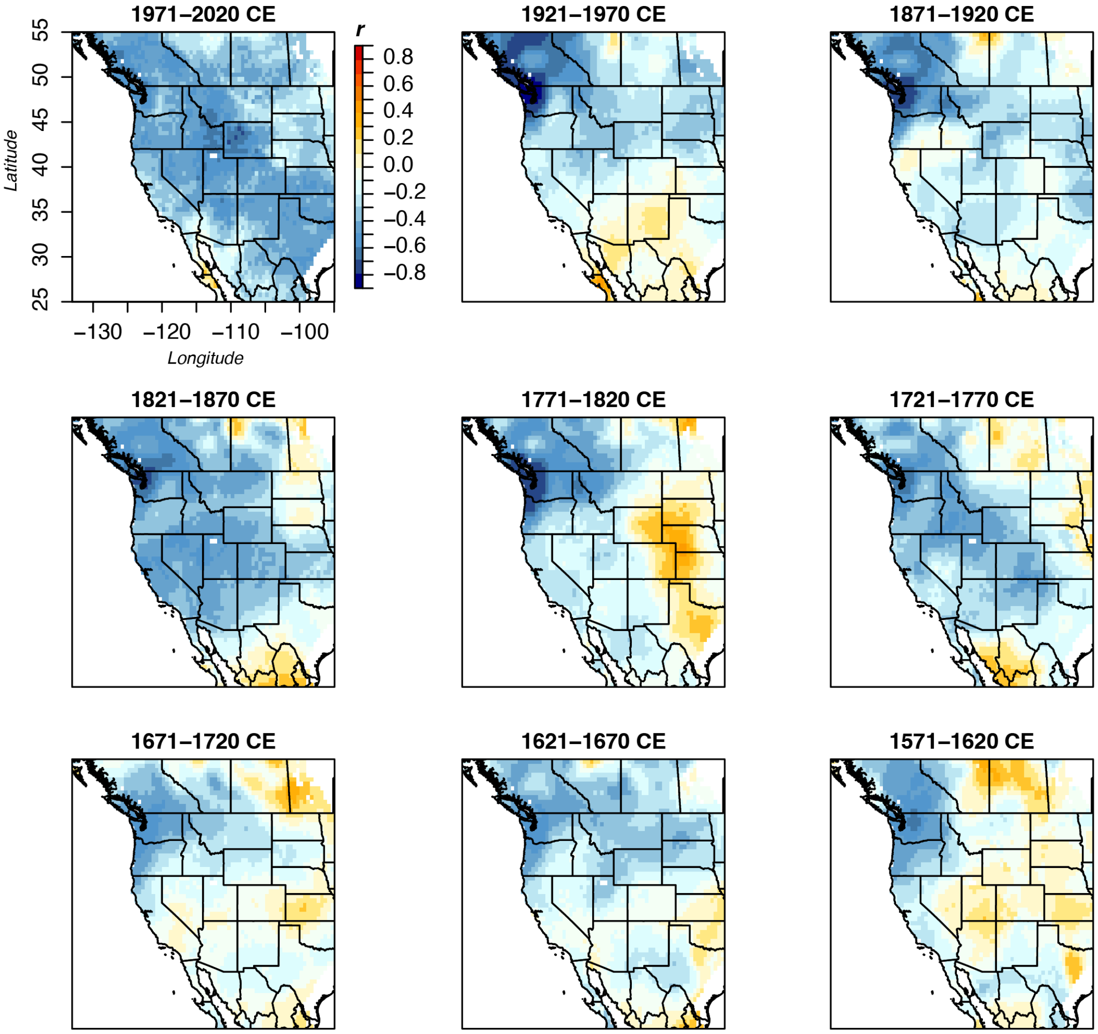

Fig. S9. Correlation between 50-year average summer maximum temperature and self-calibrating Palmer's Drought Severity Index across western North America since 1571 CE. Mapped Pearson's correlation coefficient (r) between maximum temperature anomalies from the WNATA and PDSI values from the LBDA, averaged over 50-year periods back through time. Both the temperature and PDSI anomalies are calculated using the 1553-2020 CE climate normal.

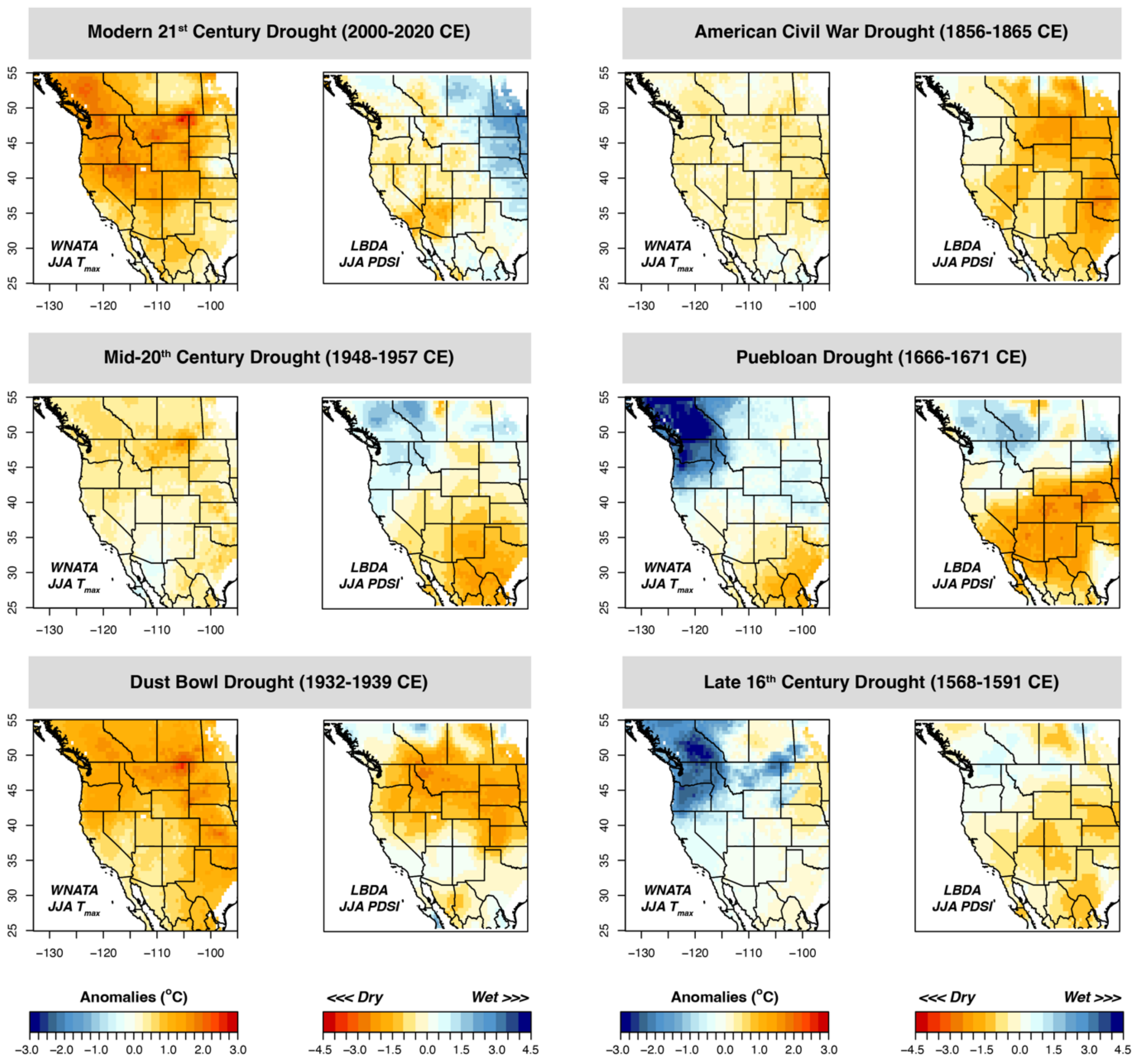

Fig. S10. Characterization of average summer maximum temperature and Palmer's Drought Severity Index across western North America during major historical drought periods since 1553 CE. Maximum temperature anomalies from the WNATA and PDSI values from the LBDA are mapped, each of which have been averaged over each historical period of exceptional drought. Both the temperature and PDSI anomalies are calculated using the 1553-2020 CE climate normal.

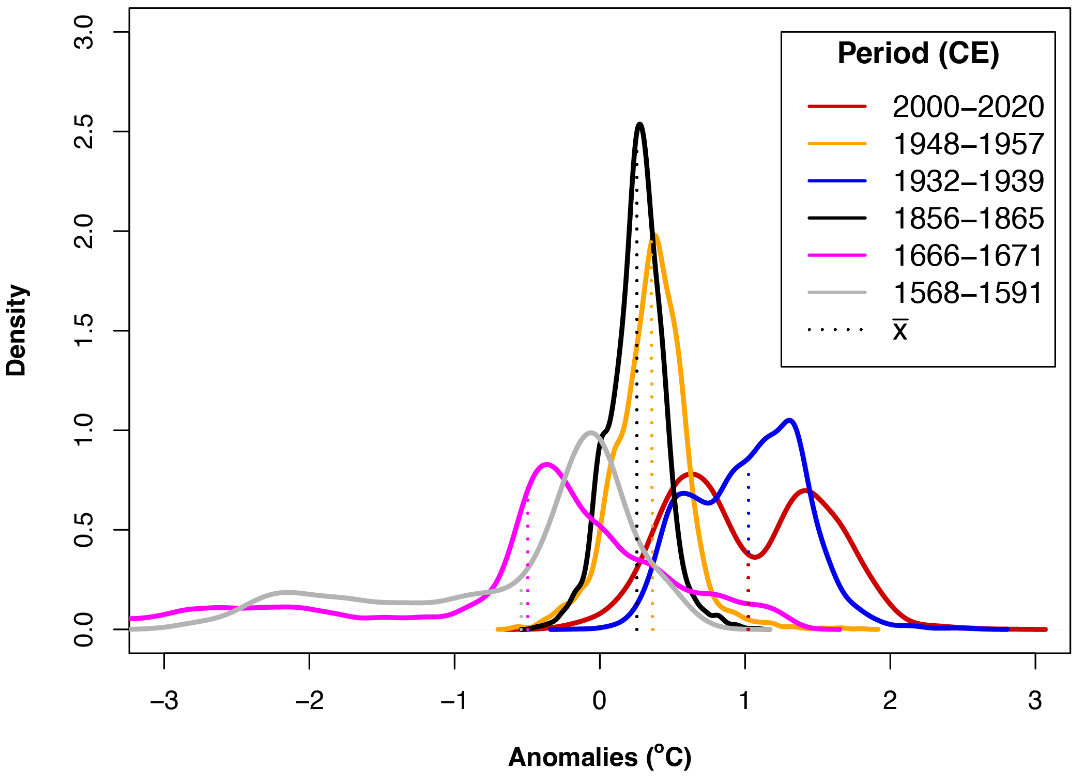

Fig. S11. Kernel density distribution of average reconstructed JJA T_{max} anomalies for multiple historical megadrought periods across Western North America. Temperature anomalies for each grid point are calculated from the WNATA, relative to the full period (1553-2020 CE).

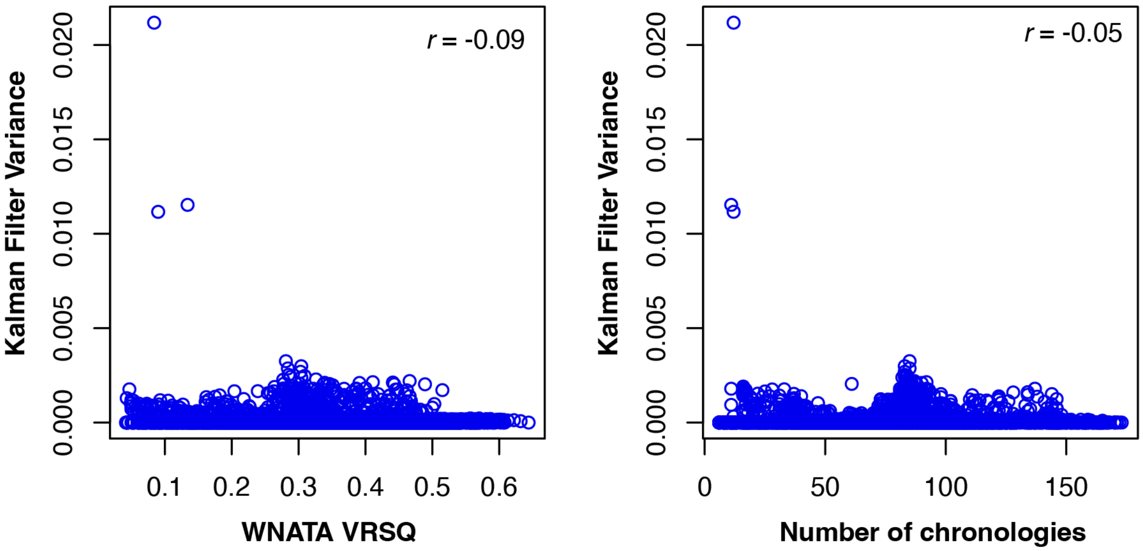

Fig. S12. No significant (p<0.01), statistical relationship between the Kalman Filter variance and the skill or number of input chronologies of each WNATA grid point reconstruction. The Kalman Filter variances between the LBDA and WNATA are plotted on the y-axis, the WNATA verification period r^2 (VRSQ) and the number of chronology predictors retained for each WNATA grid point reconstruction on the x-axis, and the Pearson's correlation coefficients between each respective pair of variables.

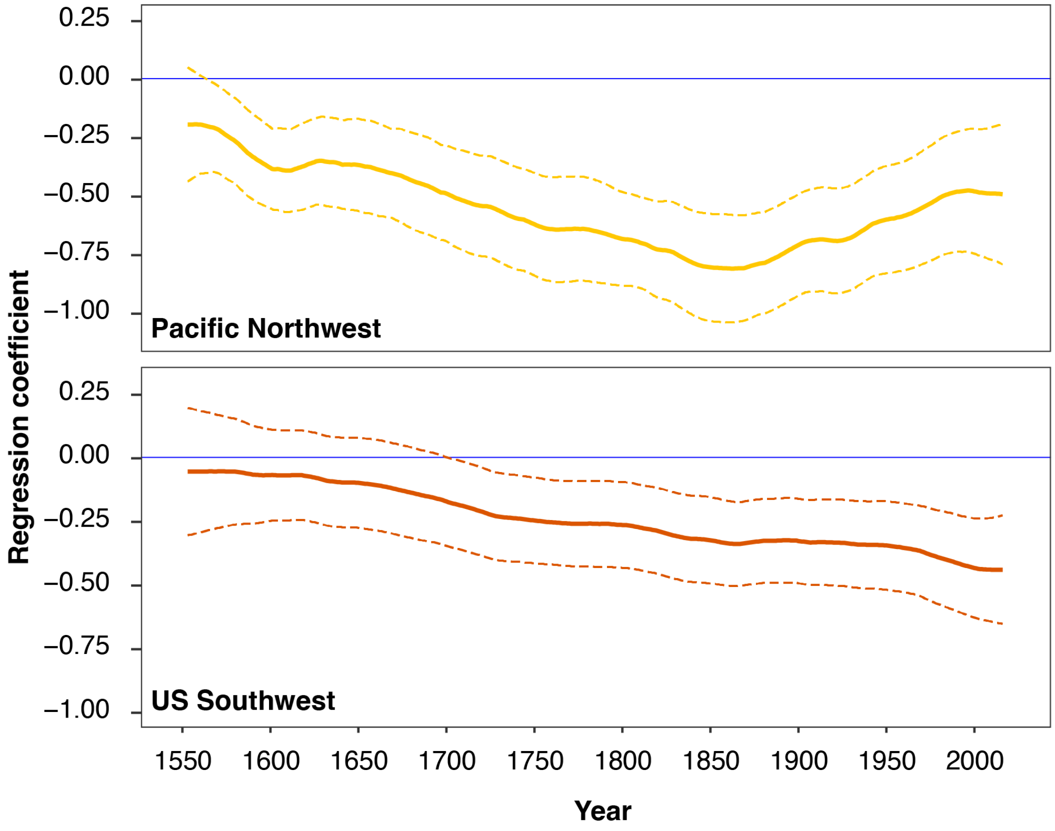

Fig. S13. Average Kalman filter traces for the WNATA and LBDA over the Pacific Northwest and US Southwest regions with no applied prefiltering. The regional average of the Kalman Filter traces or the Pacific Northwest and the United States Southwest regions, where no prefiltering was applied to the LBDA and WNATA prior to the Kalman filtering. The regression coefficients (solid, colored lines) are plotted with 95% confidence intervals (dashed, colored lines).

REFERENCES AND NOTES

1. A. AghaKouchak, L. Cheng, O. Mazdiyasni, A. Farahmand, Global warming and changes in risk of concurrent climate extremes: Insights from the 2014 California drought. *Geophys. Res. Lett.* **41**, 8847–8852 (2014).

2. Z. Hao, F. Hao, V. P. Singh, X. Zhang, Changes in the severity of compound drought and hot extremes over global land areas. *Environ. Res. Lett.* **13**, 124022 (2018).

3. A. Sarhadi, M. C. Ausín, M. P. Wiper, D. Touma, N. S. Diffenbaugh, Multidimensional risk in a nonstationary climate: Joint probability of increasingly severe warm and dry conditions *Sci. Adv.* **4**, eaau3487 (2018).

4. M. R. Alizadeh, J. Adamowski, M. R. Nikoo, A. AghaKouchak, P. Dennison, M. Sadegh, A century of observations reveals increasing likelihood of continental-scale compound dry-hot extremes. *Sci. Adv.* **6**, eaaz4571 (2020).

5. J. Zscheischler, S. Westra, B. J. Van Den Hurk, S. I. Seneviratne, P. J. Ward, A. Pitman, A. AghaKouchak, D. N. Bresch, M. Leonard, T. Wahl, X. Zhang, Future climate risk from compound events. *Nat. Clim. Change* **8**, 469–477 (2018).

6. A. AghaKouchak, F. Chiang, L. S. Huning, C. A. Love, I. Mallakpour, O. Mazdiyasni, H. Moftakhari, S. M. Papalexiou, E. Ragno, M. Sadegh, Climate extremes and compound hazards in a warming world. *Annu. Rev. Earth Planet. Sci.* **30**, 519–548 (2020).

7. J. T. Overpeck, The challenge of hot drought. *Nature* **503**, 350–351 (2013).

8. D. Griffin, K. J. Anchukaitis, How unusual is the 2012–2014 California drought? *Geophys. Res. Lett.* **41**:9017–9023 (2014).

9. F. Chiang, P. Greve, O. Mazdiyasni, Y. Wada, A. AghaKouchak, Intensified likelihood of concurrent warm and dry months attributed to anthropogenic climate change. *Water Resour. Res.* **58**, 2021WR030411 (2022).

10. A. P. Williams, E. R. Cook, J. E. Smerdon, B. I. Cook, J. T. Abatzoglou, K. Bolles, S. H. Baek, A. M. Badger, B. Livneh, Large contribution from anthropogenic warming to an emerging North American megadrought. *Science* **368**, 314–318 (2020).

11. J. Zscheischler, S. I. Seneviratne, Dependence of drivers affects risks associated with compound events. *Sci. Adv.* **3**, e1700263 (2017).

12. K. Marvel, B. I. Cook, C. J. Bonfils, P. J. Durack, J. E. Smerdon, A. P. Williams, Twentieth-century hydroclimate changes consistent with human influence. *Nature* **569**, 59–65 (2019).

13. K. J. Allen, D. C. Verdon-Kidd, J. Z. Sippo, P. J. Baker, Compound climate extremes driving recent sub-continental tree mortality in northern Australia have no precedent in recent centuries. *Sci. Rep.* **11**, 18337 (2021).

14. S. I. Seneviratne, X. Zhang, M. Adnan, W. Badi, C. Dereczynski, A. Di Luca, S. Ghosh, I. Iskander, Weather and Climate Extreme Events in a Changing Climate (Chapter 11) in *IPCC 2021: Climate Change 2021: The Physical Science Basis. Contribution of Working Group I to the Sixth Assessment Report of the Intergovernmental Panel on Climate Change*, V. Masson-Delmotte, P. Zhai, A. Pirani, S.L. Connors, C. Péan, S. Berger, N. Caud, Y. Chen Eds. (Cambridge Univ. Press, 2021) pp. 1513–1766.

15. D. R. Easterling, T. W. Wallis, J. H. Lawrimore, R. R. Heim Jr, Effects of temperature and precipitation trends on U.S. drought. *Geophys. Res. Lett.* **34**, GL031541 (2007).

16. N. S. Diffenbaugh, D. L. Swain, D. Touma, Anthropogenic warming has increased drought risk in California. *Proc. Natl. Acad. Sci.* **112**, 3931–3936 (2015).

17. O. Mazdiyasni, A. AghaKouchak, Substantial increase in concurrent droughts and heatwaves in the United States. *Proc. Natl. Acad. Sci.* **112**, 11484–11489 (2015).

18. J. T. Overpeck, B. Udall, Climate change and the aridification of North America. *Proc. Natl. Acad. Sci.* **117**,11856–11858 (2020).

19. K. A. McKinnon, A. Poppick, I. R. Simpson, Hot extremes have become drier in the United States Southwest. *Nat. Clim. Change* **11**, 598–604 (2021).

20. F. Lehner, C. Deser, I. R. Simpson, L. Terray, Attributing the U.S. Southwest's recent shift into drier conditions. *Geophys. Res. Lett.* **45**, 6251–6261 (2018).

21. B. I. Cook, J. E. Smerdon, E. R. Cook, A. P. Williams, K. J. Anchukaitis, J. S. Mankin, K. Allen, L. Andreu-Hayles, T. R. Ault, S. Belmecheri, S. Coats, Megadroughts in the Common era and the Anthropocene. *Nat. Rev. Earth Environ.* **3**, 741–757 (2022).

22. R. Seager, M. Ting, P. Alexander, J. Nakamura, H. Liu, C. Li, I. R. Simpson, Mechanisms of a meteorological drought onset: Summer 2020 to spring 2021 in southwestern North America. *J. Climate* **35**, 3767–3785 (2022).

23. B. I. Cook, R. L. Miller, R. Seager, Amplification of the North American “Dust Bowl” drought through human-induced land degradation. *Proc. Natl. Acad. Sci.* **106**, 4997–5001 (2009).

24. A. P. Williams, B. I. Cook, J. E. Smerdon, Rapid intensification of the emerging southwestern North American megadrought in 2020–2021. *Nat. Clim. Change* **12**, 232–234 (2022).

25. E. R. Cook, R. Seager, M. A. Cane, D. W. Stahle, North American drought: Reconstructions, causes, and consequences. *Earth-Sci. Rev.* **81**, 93–134 (2007).

26. S. Zhou, A. P. Williams, A. M. Berg, B. I. Cook, Y. Zhang, S. Hagemann, S. R. Lorenz, S. I. Seneviratne, P. Gentine, Land–atmosphere feedbacks exacerbate concurrent soil drought and atmospheric aridity. *Proc. Natl Acad. Sci.* **116**, 18848–18853 (2019).

27. R. Seager, A. Hooks, A. P. Williams, B. Cook, J. Nakamura, N. Henderson, Climatology, variability, and trends in the U.S. vapor pressure deficit, an important fire-related meteorological quantity. *J. Appli. Meteorol. Climatol.* **54**, 1121–1141 (2015).

28. E. R. Wahl, E. Zorita, H. F. Diaz, A. Hoell. Southwestern United States drought of the 21st century presages drier conditions into the future. *Commun. Earth Environ.* **3**, 202 (2022).

29. A. P. Williams, R. Seager, J. T. Abatzoglou, B. I. Cook, J. E. Smerdon, E. R. Cook, Contribution of anthropogenic warming to California drought during 2012–2014. *Geophys. Res. Lett.* **42**, 6819–6828 (2015).

30. PAGES 2k Consortium, A global multiproxy database for temperature reconstructions of the Common era. *Scientific Data* **4**: 1–33 (2017).

31. K. J. Anchukaitis, R. Wilson, K. R. Briffa, U. Büntgen, E. R. Cook, R. D'Arrigo, N. Davi, J. Esper, D. Frank, B. E. Gunnarson, Last millennium Northern Hemisphere summer temperatures from tree rings: Part II, spatially resolved reconstructions. *Quater. Sci. Rev.* **163**, 1–22 (2017).

32. J. T. Martin, G. T. Pederson, C. A. Woodhouse, E. R. Cook, G. J. McCabe, K. J. Anchukaitis, E. K. Wise, P. J. Erger, L. Dolan, M. McGuire, Increased drought severity tracks warming in the United States' largest river basin. *Proc. Natl. Acad. Sci.* **117**, 11328–11336 (2020).

33. K. J. Anchukaitis, J. E. Smerdon, Progress and uncertainties in global and hemispheric temperature reconstructions of the Common era. *Quater. Sci. Rev.* **286**, 107537 (2022).

34. E. R. Wahl, J. E. Smerdon, Comparative performance of paleoclimate field and index reconstructions derived from climate proxies and noise-only predictors. *Geophys. Res. Lett.* **39** (2012).

35. V. Trouet, H. F. Diaz, E. R. Wahl, A. E. Viau, R. Graham, N. Graham, E. R. Cook, A 1500-year reconstruction of annual mean temperature for temperate North America on decadal-to-multidecadal time scales. *Environ. Res. Lett.* **8**, 024008 (2013).

36. E. R. Wahl, H. F. Diaz, J. E. Smerdon, C. M. Ammann, Late winter temperature response to large tropical volcanic eruptions in temperate western North America: Relationship to ENSO phases. *Global Planet. Change* **122**, 238–250 (2014).

37. E. R. Cook, R. Seager, R. R. Heim Jr, R. S. Vose, C. Herweijer, C. Woodhouse, Megadroughts in North America: Placing IPCC projections of hydroclimatic change in a long-term palaeoclimate context. *J. Quater. Sci.* **25**, 48–61 (2010a).

38. D. W. Stahle, E. R. Cook, M. K. Cleaveland, M. D. Therrell, D. M. Meko, H. D. Grissino-Mayer, E. Watson, B. H. Luckman, Tree-ring data document 16th century megadrought over North America. *Eos. Trans. AGU* **81**, 121–125 (2000).

39. D. W. Stahle, J. S. Dean, North American tree rings, climatic extremes, and social disasters in *Dendroclimatology* (Springer, 2011), pp. 297–327.

40. D. W. Stahle, E. R. Cook, D. J. Burnette, M. C. A. Torbenson, I. M. Howard, D. Griffin, J. Villanueva Diaz, B. I. Cook, A. P. Williams, E. Watson, D. J. Sauchyn, N. Pederson, G. T. Pederson, D. Meko, B. Coulthard, C. J. Crawford, Dynamics, variability, and change in seasonal precipitation reconstructions for North America. *J. Climate* **33**, 3173–3195 (2020).

41. J. Björklund, G. von Arx, D. Nievergelt, R. Wilson, J. Van den Bulcke, B. Günther, N.J. Loader, M. Rydval, P. Fonti, T. Scharnweber, L. Andreu-Hayles, Scientific merits and analytical challenges of tree-ring densitometry. *Rev. Geophys.* **57**, 1224–1264 (2019).

42. E. R. Cook, D. M. Meko, D. W. Stahle, M. K. Cleaveland, Drought reconstructions for the continental United States. *J. Climate* **12**, 1145–1162 (1999).

43. K. R. Briffa, P. Jones, F. Schweingruber, Tree-ring density reconstructions of summer temperature patterns across western North America since 1600. *J. Climate* **5**, 735–754 (1992).

44. W. C. Palmer, *Meteorological Drought Weather Bureau Research Paper 45*, (U.S. Department of Commerce, 1965).

45. K. J. Heeter, G. L. Harley, J. T. Abatzoglou, K. J. Anchukaitis, E. R. Cook, B. L. Coulthard, L. A. Dye, I. K. Homfeld, Unprecedented 21st century heat across the Pacific Northwest of North America *Science* **6**, 5 (2023).

46. H. Visser, J. Molenaar, Kalman filter analysis in dendroclimatology. *Biometrics* **44**, 929–940 (1988).

47. B. I. Cook, J. S. Mankin, K. J. Anchukaitis, Climate change and drought: From past to future. *Current Clim. Change Rep.* **4**, 164–179 (2018).

48. C. A. Woodhouse, G. T. Pederson, K. Morino, S. A. McAfee, G. J. McCabe, Increasing influence of air temperature on upper Colorado River streamflow. *Geophys. Res. Lett.* **43**, 2174–2181 (2016).

49. B. I. Cook, T. R. Ault, J.E. Smerdon JE, Unprecedented 21st century drought risk in the American Southwest and Central Plains *Sci. Adv.* **1**, e1400082 (2015).

50. S. Bartusek, K. Kornhuber, M. Ting, 2021 North American heatwave amplified by climate change-driven nonlinear interactions. *Nat.Clim. Change* **12**, 1–8 (2022).

51. P. W. Mote, S. Li, D. P. Lettenmaier, M. Xiao, R. Engel, Dramatic declines in snowpack in the western *US Science* **1**, 2 (2018).

52. G. L. Harley, R. S. Maxwell, B. A. Black, M. F. Bekker, A multi-century, tree-ring-derived perspective of the North Cascades (USA) 2014–2016 snow drought. *Clim. Change* **162**, 127–143 (2020).

53. L. A. Dye, B. L. Coulthard, B. Hatchett, I. K. Homfeld, T. N. Salazar, J. S. Littell, K.J. Anchukaitis KJ, The severity of the 2014-2015 snow drought in the Oregon Cascades in a multicentury context. *Water Resour. Res.*, **59** (2023).

54. F. Babst, O. Bouriaud, B. Poulter, V. Trouet, M. P. Girardin, D. C. Frank, Twentieth century redistribution in climatic drivers of global tree growth *Sci. Adv.* **5**, eaat4313 (2019).

55. J. M. Denissen, A. J. Teuling, A. J. Pitman, S. Koirala, M. Migliavacca, W. Li, M. Reichstein, A. J. Winkler, C. Zhan, R. Orth, Widespread shift from ecosystem energy to water limitation with climate change. *Nat. Clim. Change* **12**, 677–684 (2022).

56. N. A. Houston, S. L. Gonzales-Bradford, A. T. Flynn, S. L. Qi, S. M. Peterson, J. S. Stanton, D. W. Ryter, T. L. Sohl, G. B. Senay, *Geodatabase Compilation of Hydrogeologic, Remote Sensing, and Water-Budget-Component Data for the High Plains Aquifer, 2011* (U.S. Geological Survey Data Series 777:12, 2013).

57. S. B. Roy, L. Chen, E. H. Girvetz, E. P. Maurer, W. B. Mills, T. M. Grieb, Projecting water withdrawal and supply for future decades in the US under climate change scenarios. *Environ. Sci. Technol.* **6**, 2545–2556 (2012).

58. B. Udall, J. Overpeck, The twenty-first century Colorado River hot drought and implications for the future. *Water Resour. Res.* **53**, 2404–2418 (2017).

59. N. J. Rosenberg, D. J. Epstein, D. Wang, L. Vail, R. Srinivasan, J. G. Arnold, Possible impacts of global warming on the hydrology of the Ogallala aquifer region. *Clim. Change* **42**, 677–692 (1999).

60. K. E. Trenberth, A. Dai, G. Van Der Schrier, P. D. Jones, J. Barichivich, K. R. Briffa, J. Sheffield, Global warming and changes in drought. *Nat. Clim. Change* **4**, 17–22 (2014).

61. H. Douville, K. Raghavan, J. Renwick, R. P. Allan, P. A. Arias, M. Barlow, R. Cerezo-Mota, A. Cherchi, T. Y. Gan, J. Gergis, D. Jiang, Water cycle changes in *Climate Change 2021: The Physical Science Basis. Contribution of Working Group I to the Sixth Assessment Report of the Intergovernmental Panel on Climate Change*, Masson-Delmotte V, Zhai P, Pirani A, Connors SL, Péan C, Berger S, *et al.* Eds. (Cambridge Univ. Press, 2021), pp. 1055–1210.

62. R. Seager, N. Henderson, M. A. Cane, H. Liu, J. Nakamura, Is there a role for human-induced climate change in the precipitation decline that drove the California drought? *J. Climate* **30**, 10237–10258 (2017).

63. R. Seager, M. Ting, P. Alexander, H. Liu, J. Nakamura, C. Li, M. Newman, Ocean-forcing of cool season precipitation drives ongoing and future decadal drought in southwestern North *America Science* **6**,141 (2023).

64. K. P. Tripathy, S. Mukherjee, A. K. Mishra, M. E. Mann, A. P. Williams, Climate change will accelerate the high-end risk of compound drought and heatwave events. *Proc. Natl. Acad. Sci.* **120**, e2219825120 (2023).

65. G. van der Schrier, J. Barichivich, K. R. Briffa, A scPDSI-based global data set of dry and wet spells for 1901-2009. *J. Geophys. Res. Atmosp.* **118**, 4025–4048 (2013).

66. J. Barichivich, T. J. Osborn, I. Harris, G. van der Schrier, P. D. Jones, Drought [in "State of the Climate in 2019"]. *Bull. Am. Meteorol. Soc.* **101**, S1-S429 (2020).

67. D. McCarroll, E. Pettigrew, A. Luckman, F. Guibal, J.L. Edouard, Blue reflectance provides a surrogate for latewood density of high-latitude pine tree rings. *Arctic, Antarctic, Alpine Res.* **34**, 450–453 (2002).

68. F. Schweingruber, H. Fritts, O. Bräker, L. Drew, E. Schär, The X-ray technique as applied to dendroclimatology. *Dendrochronologia* **42**, 42–50 (1978)

69. R. Wilson, K. Anchukaitis, L. Andreu-Hayles, E. Cook, R. D'Arrigo, N. Davi, L. Haberbauer, P. Krusic, B. Luckman, D. Morimoto, R. Oelkers, Improved dendroclimatic calibration using blue intensity in the southern Yukon. *Holocene* **29**, 1817–1830 (2019).

70. K. J. Heeter, G. L. Harley, J. T. Maxwell, R. J. Wilson, J. T. Abatzoglou, S. A. Rayback, M. L. Rochner, K. A. Kitchens, Summer temperature variability since 1730 CE across the low-to-mid latitudes of western North America from a tree ring blue intensity network. *Quater. Sci. Rev.* **267**, 107064 (2021a).

71. T. M. Melvin, K. R. Briffa, K. Nicolussi, M. Grabner, Time-varying-response smoothing. *Dendrochronologia* **25**, 65–69 (2007).

72. T. M. Melvin, K. R. Briffa, A 'signal-free' approach to dendroclimatic standardisation. *Dendrochronologia* **26**, 71–86 (2008).

73. E. R. Cook, K. J. Anchukaitis, B. M. Buckley, R. D. D'Arrigo, G. C. Jacoby, W. E. Wright, Asian monsoon failure and megadrought during the last millennium. *Science* **328**: 486–489 (2010b).

74. E. R. Cook, P. J. Krusic, K. J. Anchukaitis, B. M. Buckley, T. Nakatsuka, M. Sano, Tree-ring reconstructed summer temperature anomalies for temperate East Asia since 800 C.E. *Climate Dynam.* **41**, 2957–2972 (2013).

75. E. R. Cook, R. Seager, Y. Kushnir, K. R. Briffa, U. Büntgen, D. Frank, P. J. Krusic, W. Tegel, G. van der Schrier, L. Andreu-Hayles, M. Baillie, Old World megadroughts and pluvials during the Common era. *Sci. Adv.* **1**, e1500561 (2015).

76. H. Fritts, *Tree rings and climate* (Elsevier, 1976).

77. E. R. Cook, K. R. Briffa, P. D. Jones, Spatial regression methods in dendroclimatology: A review and comparison of two techniques. *Int. J. Climatol.* **14**, 379–402 (1994).

78. G. R. North, T. L. Bell, R. F. Cahalan, Sampling errors in the estimation of empirical orthogonal functions. *Mon. Weather Rev.* **110**, 699–706 (1982).

79. A. C. Harvey, *Forecasting, structural time series models and the Kalman filter* (Cambridge Univ. Press, Cambridge, 1990).

80. E. R. Cook, A. H. Johnson, Climate change and forest decline: A review of the red spruce case. *Water Air Soil Pollut.* **48**, 127–140 (1989).

81. G. C. Jacoby, R. D. D'Arrigo, Tree ring width and density evidence of climatic and potential forest change in Alaska. *Global Biogeochem. Cycles* **9**, 227–234 (1995).

82. K. J. Allen, R. Villalba, A. Lavergne, J. G. Palmer, E. C. Cook, P. Fenwick, D. M. Drew, C. S. M. Turney, P. J. Baker, A comparison of some simple methods used to detect unstable temperature responses in tree-ring chronologies. *Dendrochronologia* **48**, 52–73 (2018).

83. C.M. Hurvich, C-L. Tsai, Regression and time series model selection in small samples. *Biometrika* **76**, 297–307 (1989).

84. R. H. Jones, Time series analysis- Time domain in *Probability, Statistics, and Decision Making in the Atmospheric Sciences*, A. H. Murphy, R. W. Katz, Eds. (Westview Press, 1985), pp. 223–259.

85. S.S. George, T.R. Ault, The imprint of climate within Northern Hemisphere trees. *Quater. Sci. Rev.* **89**,1–4 (2014).

86. R. Wilson, K. Anchukaitis, K.R. Briffa, U. Büntgen, E. Cook, R. D’arrigo, N. Davi, J. Esper, D. Frank, B. Gunnarson, G. Hegerl, Last millennium Northern Hemisphere summer temperatures from tree rings: Part I: The long term context. *Quater. Sci. Rev.* **134**,1–18 (2016).
87. P.R. Sheppard, L.J. Graumlich, L.E. Conkey, Reflected-light image analysis of conifer tree rings for reconstructing climate. *Holocene* **6**, 62–68 (1996).
88. F. Schweingruber, K. Briffa, P. Nogler, A tree-ring densitometric transect from Alaska to Labrador. *Int. J. Biometeorol.* **37**, 151–169 (1993).
89. F. H. Schweingruber, K. R. Briffa, Tree-ring density networks for climate reconstruction in *Climatic variations and forcing mechanisms of the last 2000 years* (Springer, 1996), pp. 43–66.
90. B. H. Luckman, K. R. Anchu, P. Jones, F. Schweingruber, Tree-ring based reconstruction of summer temperatures at the Columbia Icefield, Alberta, Canada, AD 1073-1983. *Holocene* **7**, 375–389 (1997).
91. R. J. Wilson, B. H. Luckman, Dendroclimatic reconstruction of maximum summer temperatures from upper treeline sites in Interior British Columbia, Canada, *Holocene* **13**, 851–861 (2003).
92. B. Luckman, R. Wilson, Summer temperatures in the Canadian Rockies during the last millennium: A revised record. *Climate Dynam.* **24**,131–144 (2005).
93. K. J. Anchukaitis, R. D. D’Arrigo, L. Andreu-Hayles, D. Frank, A. Verstege, A. Curtis, B. M. Buckley, G. C. Jacoby, E. R. Cook, Tree-ring-reconstructed summer temperatures from northwestern North America during the last nine centuries. *J. Climate* **26**, 3001–3012 (2013).
94. R. Wilson, R. Rao, M. Rydval, C. Wood, L. Å. Larsson, B. H. Luckman, Blue Intensity for dendroclimatology: The BC blues: A case study from British Columbia, Canada, *Holocene* **24**:1428–1438 (2014).
95. G. Wiles, J. Charlton, R. J. Wilson, R. DArrigo, B. Buma, J. Krapek, B. V. Gaglioti, N. Wiesenberg, R. Oelkers, Yellow-cedar blue tntensity tree ring chronologies as records of climate, Juneau, Alaska, USA. *Canadian J. Forest Res.* **49**, 1483, 1492 (2019).

96. K. J. Heeter, G. L. Harley, J. T. Maxwell, J. H. McGee, T. J. Matheus, Late summer temperature variability for the Southern Rocky Mountains (USA) since 1735 CE: Applying blue light intensity to low-latitude Picea engelmannii Parry ex Engelm. *Clim. Change* **162**, 965–988 (2020).

97. K. J. Heeter, M. L. Rochner, G. L. Harley, Summer air temperature for the Greater Yellowstone Ecoregion (770–2019 CE) over 1,250 years. *Geophys. Res. Lett.* **48**, e2020GL092269 (2021b).

98. K. R. Briffa, T. J. Osborn, F. H. Schweingruber, I. C. Harris, P. D. Jones, S. G. Shiyatov, E. A. Vaganov, Low-frequency temperature variations from a northern tree ring density network. *J. Geophys. Res. Atmos.* **106**, 2929–2941 (2001).

99. F. Wang, D. Arseneault, É. Boucher, F. Gennaretti, S. Yu, T. Zhang, Tropical volcanoes synchronize eastern Canada with Northern Hemisphere millennial temperature variability. *Nat. Commun.* **13**, 1–10 (2022).

100. M. Rydval, L. Å. Larsson, L. McGlynn, B. E. Gunnarson, N. J. Loader, G. H. Young, R. Wilson, Blue intensity for dendroclimatology: Should we have the blues? Experiments from Scotland *Dendrochronologia* **32**, 191–204 (2014).

101. J. Esper, E. Düthorn, P. J. Krusic, M. Timonen, U. Büntgen, Northern European summer temperature variations over the Common Era from integrated tree-ring density records. *J. Quater. Sci.* **29**, 487–494 (2014).

102. M. Rydval, D. L. Druckenbrod, M. Svoboda, V. Trotsiuk, P. Janda, M. Mikoláš, V. Čăda, R. Băce, M. Teodosiu, R. Wilson, Influence of sampling and disturbance history on climatic sensitivity of temperature-limited conifers. *Holocene* **28**, 1574–1587 (2018).

103. L. J. Lücke, G. C. Hegerl, A. P. Schurer, R. Wilson R, Effects of memory biases on variability of temperature reconstructions. *J. Climate* **32**, 8713–8731 (2019).

104. R. J. Kaczka, R. Wilson, I-BIND: International Blue Intensity Network Development Working Group. *Dendrochronologia* **68**, 125859 (2021).

105. L. Larsson, CooRecorder and Cdendro programs of the CooRecorder/C-dendro package version 7.7 (2014).

106. R. L. Holmes, Computer-assisted quality control in tree-ring dating and measurement. *Tree-Ring Bulletin* **43**, 69–75 (1983).

107. K. J. Heeter, D. J. King, G. L. Harley, R. J. Kaczka, Video tutorial: Measuring blue intensity with the CooRecorder software application. *Dendrochronologia* **76**, 125999 (2022).

108. D. W. Stahle, E. R. Cook, D. J. Burnette, J. Villanueva, J. Cerano, J. N. Burns, D. Griffin, B. I. Cook, R. Acuna, M. C. Torbenson, P. Szejner, The Mexican Drought Atlas: Tree-ring reconstructions of the soil moisture balance during the late pre-Hispanic, colonial, and modern eras. *Quaternary Science Reviews* **149**, 34–60 (2016).

109. R. Touchan, K. J. Anchukaitis, D. M. Meko, M. Sabir, S. Attalah, A. Aloui, Spatiotemporal drought variability in northwestern Africa over the last nine centuries. *Climate Dynam.* **37**, 237–252 (2011).
